# Angular velocity characteristics and inter-limb asymmetry during jodan-tsuki execution in a world champion female karate athlete: a case study

**DOI:** 10.3389/fspor.2026.1917343

**Published:** 2026-10-08

**Authors:** Soyib Tajibaev, Tolibjon Arslanov, Mekhriddin Abdullayev, Aziz Utepov, Zamira Tuganboyeva, Ulbosin Kubeisinova, Davron Omonov, Abror Rejametov

**Affiliations:** 1 Uzbek State University of Physical Education and Sport, Tashkent, Uzbekistan; 2 Bukhara State University, Bukhara, Uzbekistan

**Keywords:** angular velocity, biomechanics, inter-limb asymmetry, jodan-tsuki, karate, kumite, three-dimensional motion analysis

## Abstract

**Background:**

Jodan-tsuki is a frequently performed offensive technique in kumite karate requiring rapid, coordinated movement. However, three-dimensional evidence on angular velocity characteristics and inter-limb differences during Jodan-tsuki in elite female karate athletes remains limited.

**Purpose:**

This study investigated kinematic characteristics, peak angular velocity profiles, and inter-limb differences during right-hand Jodan-tsuki in a world champion female karate athlete using three-dimensional motion analysis.

**Methods:**

In this case study, a world champion female karate athlete (20 years; 50 kg weight category) performed 22 technically successful right-hand Jodan-tsuki trials. Three-dimensional motion capture was used to quantify trunk, cervical, upper- and lower-extremity kinematics. Temporal-spatial variables, joint range of motion (ROM), peak joint and segment angular velocities, and bilateral asymmetry indices (AI) were calculated. Descriptive statistics, coefficients of variation, and 95% confidence intervals were determined across trials.

**Results:**

The highest peak angular velocity was observed at the striking-side elbow (351.42 ± 38.12°·s⁻¹), followed by the right ankle (237.53 ± 24.02°·s⁻¹), left knee (174.39 ± 18.20°·s⁻¹), and right shoulder abduction–adduction (168.45 ± 18.20°·s⁻¹). Peak magnitudes differed across the analysed joint and segment variables, with the largest whole-cycle absolute peak at the striking-side elbow. Peak timing was not quantified; therefore, proximal-to-distal sequencing was not demonstrated. Greater task-related bilateral differences were observed for shoulder flexion (AI = 106.2%), elbow flexion (AI = 88.0%), and hip flexion (AI = 78.3%), whereas smaller differences were observed for knee flexion (AI = 9.8%) and ankle dorsiflexion–plantarflexion (AI = 10.1%). Trunk angular ranges were smaller than cervical angular ranges.

**Conclusions:**

In this world champion female karate athlete, right-hand Jodan-tsuki was characterized by the highest whole-cycle peak angular velocity at the striking-side elbow and task-related bilateral differences of varying numerical magnitude. Peak angular velocity magnitudes describe the distribution of whole-cycle peak values but do not establish their temporal order. These findings provide a three-dimensional biomechanical characterization of Jodan-tsuki in this individual elite athlete. Given the single-athlete case-study design, the findings should be interpreted as an individual biomechanical profile rather than generalized to elite karate athletes.

## 1 Introduction

Karate kumite is a highly dynamic combat sport in which successful performance depends on the rapid execution of offensive and defensive techniques, precise intersegmental coordination, and effective force transmission throughout the body (1). Among the various punching techniques used in competitive karate, Jodan-tsuki represents an important scoring action directed toward the opponent's head region and requires the integration of lower-limb force generation, trunk rotation, upper-limb acceleration, and visual control to achieve maximum effectiveness (2, 3).

Previous biomechanical investigations have described karate punching as a multisegmental action involving coordinated contributions from the lower extremities, trunk, and upper limbs. Gulledge and Dapena (2) reported coordinated contributions of multiple body segments during punching actions, while Vences Brito et al. (4) described kinematic and electromyographic characteristics associated with karate punching. Rinaldi et al. (5) further examined punch velocity, timing, and other performance-related indices during Junzuki execution, highlighting the relevance of movement organization to the biomechanical analysis of karate punching. In addition, Martinez de Quel and Bennett (6) demonstrated measurable kinematic differences between self-initiated and reactive karate punches performed in response to an external stimulus. This distinction is relevant to the interpretation of the present study because Jodan-tsuki was performed as a standardized non-contact action into free space rather than in response to a moving opponent or external reactive stimulus.

Advances in motion-capture technology have enabled increasingly detailed investigations of martial arts techniques. Hachaj et al. (7) demonstrated the usefulness of three-dimensional motion-capture systems for analyzing highly skilled karate movements, whereas Szczęsna et al. (8) provided comprehensive optical motion-capture datasets for selected karate techniques. More recently, Vuković et al. (9) confirmed the reliability of modern kinematic sensor systems for evaluating reverse-punch performance in karate athletes. Collectively, these studies support the application of three-dimensional biomechanical analysis for understanding movement organization and performance determinants in combat sports. More recently, reviews of wearable and optical sensing technology in combat sports have emphasized that measurement validity depends on the whole measurement system - sensor or marker type, attachment method, and processing algorithm - rather than on any single component in isolation (10), and that marker location and joint-constraint assumptions measurably affect the joint kinematics reported by a given biomechanical model (10).

One of the biomechanical principles commonly described in explosive striking actions is proximal-to-distal sequencing, in which the timing of motion across body segments contributes to the organization of the striking action (11). Such temporal organization has been examined in karate punching techniques (2), boxing punches (12, 13), taekwondo kicking techniques (14), and other combat-sport movements (15). Angular-velocity analysis can contribute to the biomechanical description of these multisegmental actions; however, peak angular-velocity magnitudes alone do not establish temporal proximal-to-distal sequencing, intersegmental momentum transfer, or kinetic-chain efficiency.

Strength and power development are recognized as important determinants of explosive sport performance, with resistance-training adaptations contributing to improved force-production capabilities (16).

Another important aspect of combat-sport biomechanics is the presence of inter-limb asymmetry. Long-term sport-specific training often results in functional asymmetries between dominant and non-dominant limbs, particularly in unilateral striking sports. Bishop et al. (17) proposed standardized approaches for quantifying inter-limb asymmetry and highlighted methodological considerations related to asymmetry calculation. Subsequently, Bishop et al. (18) demonstrated through a systematic review that the influence of asymmetry on athletic performance is task-specific and may vary according to the physical and technical demands of the sport. Despite the growing interest in asymmetry research, limited information is available regarding asymmetry patterns during elite karate punching techniques, particularly in female athletes competing at the highest international level. This gap persists even in the most recent karate-specific literature: a 2025 study of sixty-one elite WKF kumite athletes assessing inter-limb strength asymmetry via isometric testing explicitly noted that its findings could not indicate which movement pattern (punching versus kicking) underlay the observed asymmetries, and that sex and weight category were not evaluated as differentiating factors (19). This underscores that existing karate asymmetry research has primarily addressed isometric strength rather than technique-specific three-dimensional kinematic asymmetry, and has not isolated female athletes at the highest competitive level as a distinct focus - the specific gap the present case study addresses.

Elite-athlete case studies have become an established methodological approach in sports biomechanics because access to world-class performers is inherently limited (20–22). Rather than generating population-level estimates, case studies provide detailed descriptions of movement strategies and performance characteristics in exceptional athletes. This approach has been successfully applied in karate (23), boxing (24), mixed martial arts (25), handcycling (26), and other high-performance sporting contexts. Such investigations provide valuable benchmark data that may not be captured through traditional group-based analyses. However, this benefit comes with an inherent trade-off in generalizability: findings from a single exceptional athlete describe that athlete's own solution and cannot be assumed to extend to other athletes, even within the same competitive tier, as discussed further in Section 5. Recent (2023–2026) contributions in this area include a case series in handcycling (26) and an analysis of the strength and conditioning demands specific to Kyokushin karate (27). This athlete's specific competitive record - World Champion, multiple-time Asian Champion, and International-Class Master of Sport - situates her among the population for whom a single-athlete design is most justified. Beyond karate specifically, single-athlete biomechanical case studies have been used more broadly across combat sports and related movement disciplines, including boxing, mixed martial arts, and Tai Chi push hands, to characterize movement organization, segmental coordination, and sport-specific technical adaptations in highly skilled performers (24, 25, 28–31), and karate performance in particular requires the integration of technical, neuromuscular, and physiological capacities during high-intensity combat actions (32).

Despite advances in karate biomechanics research, detailed three-dimensional evidence describing joint and segment angular-velocity characteristics, trunk and cervical kinematics, and task-related bilateral differences during Jodan-tsuki execution in world-class female karate athletes remains limited. Previous studies have examined various aspects of karate punching, including punch velocity, electromyographic activity, physiological responses, and general kinematic characteristics (2–5); however, fewer data are available describing these biomechanical characteristics simultaneously during Jodan-tsuki execution in an athlete competing at the highest international level. The present case study therefore addresses this gap through an in-depth descriptive three-dimensional characterization of Jodan-tsuki execution in a world champion female athlete, with particular emphasis on whole-cycle peak joint and segment angular-velocity magnitudes, trunk and cervical kinematics, and task-related bilateral differences. The study is intended to provide an individual biomechanical profile rather than to establish a previously undescribed biomechanical mechanism or temporal proximal-to-distal sequencing pattern.

The contribution of the present study lies in the detailed, simultaneous three-dimensional characterization of whole-cycle peak joint and segment angular-velocity magnitudes, trunk and cervical kinematics, temporal–spatial characteristics, and task-related bilateral differences during Jodan-tsuki execution in a single world champion female athlete. Previous studies have addressed three-dimensional kinematics, intersegmental coordination, and punching characteristics in karate (2, 4, 5, 7), and the present case study is therefore positioned as an in-depth extension of this literature rather than as the identification of a previously undescribed biomechanical mechanism. In particular, detailed evidence combining these biomechanical characteristics during Jodan-tsuki execution in an elite female athlete at the highest international competitive level remains limited. Furthermore, although inter-limb asymmetry has received increasing attention in combat-sport research, recent karate-specific evidence has primarily examined strength-related asymmetry rather than technique-specific three-dimensional kinematic bilateral differences (33). Accordingly, the present study provides an individual descriptive biomechanical profile of Jodan-tsuki execution while avoiding inference regarding temporal proximal-to-distal sequencing, which was not directly quantified in the present analysis.

The case-study approach was selected because access to world-class athletes is inherently limited, and detailed biomechanical profiling of exceptional performers can provide valuable insight into movement characteristics at the highest level of international competition. Given this athlete's exceptional competitive status and international achievements, a single-athlete case-study design was considered appropriate for providing a detailed biomechanical characterization of Jodan-tsuki execution in this athlete. Accordingly, the primary objective of the present study was to characterize whole-cycle peak joint and segment angular velocity magnitudes during right-hand Jodan-tsuki execution in a world champion female karate athlete. Secondary objectives were to describe task-related bilateral differences, joint range-of-motion characteristics, trunk and cervical kinematics, and selected temporal-spatial characteristics. Because the timing of individual angular-velocity peaks was not retained, the study was not designed to test temporal proximal-to-distal sequencing. It was hypothesized that peak angular velocity magnitudes would differ across the analysed anatomical regions and between the task-defined right and left sides.

## 2 Materials and methods

### 2.1 Study design and sample structure

This study employed a descriptive biomechanical case-study design. The sample structure was *N* = 1 athlete, with n_trials = 22 technically successful trials retained for analysis, all recorded within a single testing session. This notation is used throughout the Methods, Results, and table notes to make explicit that the analysed observations represent repeated trials from a single athlete rather than independent observations from a sample of athletes. Three-dimensional motion analysis was used to quantify temporal–spatial characteristics, joint range of motion (ROM), peak joint and segment angular-velocity magnitudes, trunk and cervical kinematics, and task-related bilateral differences during Jodan-tsuki execution.

### 2.2 Participant

The participant was an elite female karate athlete representing the Uzbekistan National Karate Team. At the time of testing, she was 20 years old, with a body height of 1.68 m and a body mass of 50 kg. The athlete held the title of International-Class Master of Sport of Uzbekistan and was the reigning World Champion in the women's −50 kg weight category. A summary of her competitive achievements is provided in Supplementary Table S1.

The athlete had 12 years of systematic karate training experience and, at the time of testing, trained 5–6 days per week, with individual training sessions typically lasting approximately 1.5–2 h. She reported no injuries during the preceding six months that affected training or performance. Testing was conducted approximately seven weeks after her most recent competition. The athlete performed all trials barefoot to permit consistent placement and unobstructed tracking of the lower-limb and foot markers, and testing was conducted on the laboratory floor. Standardised stance dimensions were not separately recorded; however, the athlete adopted the same initial fighting stance for all trials. All trials were completed within a single testing session, and the same marker placement was maintained throughout the session; markers were not removed or repositioned between trials.

Throughout this manuscript, the right side is referred to as the athlete's preferred, task-defined striking side because she executed Jodan-tsuki with her right hand and led with her right side during testing. No standardised handedness or limb-dominance assessment was performed. Therefore, this designation should not be interpreted as formally assessed anatomical or neurological dominance. This distinction is acknowledged as a limitation in Section 5.

### 2.3 Instrumentation and laboratory coordinate system

Three-dimensional kinematic data were collected at the Sports Biomechanics Laboratory of the Scientific Research Center, Uzbek State University of Physical Education and Sport (Chirchik, Uzbekistan), using the STT Systems 3D Motion Analysis platform (3DMA, version 2023.0; STT Systems, San Sebastián, Spain). The same software version designation (2023.0) is used consistently throughout this manuscript. Motion capture was performed using a 12-camera infrared optical system operating at a sampling frequency of 240 Hz.

Prior to the testing session, the capture volume was calibrated according to the manufacturer's recommended calibration procedure, and calibration quality was verified within the 3DMA software before data collection commenced. The numerical calibration residual/reconstruction error displayed during the original testing session and the corresponding numerical acceptance threshold were not retained in the available study records and therefore could not be reported retrospectively. This limitation is acknowledged because these values would provide additional information regarding the measurement quality of the calibrated capture volume.

#### 2.3.1 Laboratory (global) coordinate system

A single, fixed laboratory coordinate system defined within the 3DMA environment was used for the analyses reported in this manuscript. In this global reference frame, the *Y*-axis represented the vertical direction, whereas the X- and Z-axes represented the two horizontal directions. The coordinate system was laboratory-fixed rather than participant-based. Because the available study records did not retain a definitive mapping of the X- and Z-axes to participant-relative anteroposterior and mediolateral directions, these assignments are not retrospectively specified. Where directional spatial variables are reported, they are therefore described according to their biomechanical movement direction rather than by assigning an unverified anatomical direction to the X- or Z-axis. Relative joint kinematics were calculated using the segment-specific coordinate definitions implemented within the 3DMA biomechanical model, as described in Section 2.4.

### 2.4 Biomechanical model, marker configuration and model limitations

A full-body biomechanical model consisting of 19 retroreflective markers was used. Marker placement was performed by one experienced investigator according to the 19-marker configuration implemented for the 3DMA full-body analysis used in this study. The anatomical location of each marker is provided in Table 1 and illustrated in Figure 1. The model comprised the head, trunk, pelvis, upper arm, forearm, thigh, shank, and foot segments.

**Table 1:** Anatomical location of the 19 retroreflective markers of the full-body model used for three-dimensional motion capture (*N* = 1 athlete; single marker application).

No.	Anatomical landmark	Side	Segment defined
1	Forehead (frontal region)	–	Head
2	Vertex (superior cranial point)	–	Head
3	Occipital region	–	Head
4	Acromion process	Left	Trunk/upper arm
5	Acromion process	Right	Trunk/upper arm
6	Lateral epicondyle of the humerus (elbow region)	Left	Upper arm/forearm
7	Lateral epicondyle of the humerus (elbow region)	Right	Upper arm/forearm
8	Wrist region (distal forearm/hand segment)	Left	Forearm/hand
9	Wrist region (distal forearm/hand segment)	Right	Forearm/hand
10	Anterior superior iliac spine	Left	Pelvis
11	Anterior superior iliac spine	Right	Pelvis
12	Lateral femoral epicondyle (knee joint)	Left	Thigh/shank
13	Lateral femoral epicondyle (knee joint)	Right	Thigh/shank
14	Lateral malleolus (ankle joint)	Left	Shank/foot
15	Lateral malleolus (ankle joint)	Right	Shank/foot
16	Forefoot (dorsal aspect, second metatarsal region)	Left	Foot
17	Forefoot (dorsal aspect, second metatarsal region)	Right	Foot
18	C7/T1 vertebral region	–	Trunk
19	Sacral–pelvic region (posterior)	–	Pelvis

Marker numbers correspond to the labels shown in Figure 1. *N* = 1 athlete; markers were applied once and maintained throughout the single testing session.

**Figure 1:**
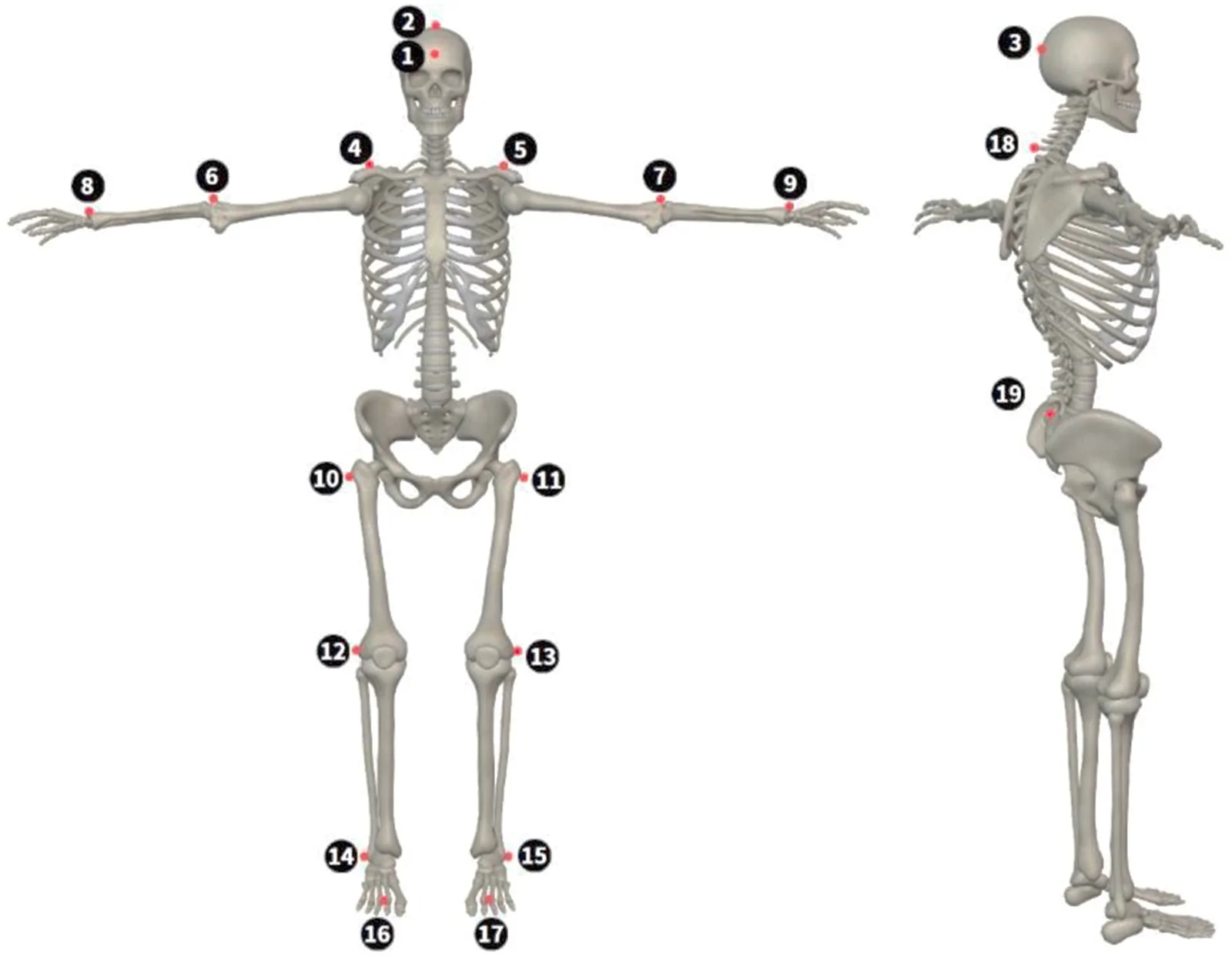
Nineteen-marker configuration used for three-dimensional motion analysis, showing the anatomical marker locations in front and side views.

#### 2.4.1 Joint centres, local coordinate systems and rotation sequences

Segment orientations, joint centres, and joint kinematics were computed automatically using the proprietary algorithms implemented within the 3DMA software environment, based on the marker configuration described above and the calibration procedures implemented within the software. The underlying computational procedures were not accessible to the investigators. Consequently, the specific regression equations or geometric procedures used to estimate joint centres, the exact definitions of the segment-specific local coordinate systems, and the Cardan/Euler rotation sequences used for joint-angle calculations could not be reported. This limitation reduces the reproducibility of the biomechanical model and should be considered when interpreting multiplanar joint measures, particularly those involving the pelvis, hip, shoulder, and cervical regions. These methodological limitations are further addressed in Section 5.

#### 2.4.2 Direction conventions

Directional signs were interpreted according to the conventions reported by the 3DMA software for each kinematic variable. For trunk and cervical motions, right lateral flexion and right axial rotation were represented as positive values, whereas left lateral flexion and left axial rotation were represented as negative values. Flexion–extension and abduction–adduction variables were interpreted according to the corresponding positive/negative directional labels provided by the 3DMA output. Because the software-specific sign conventions may vary according to the joint and movement plane, the anatomical direction of each signed value was interpreted using the corresponding 3DMA variable definition rather than assuming a universal sign convention across all joints.

#### 2.4.3 Known limitations of the marker model

This marker configuration is an anatomical-landmark model and does not include independent technical clusters on the pelvis, thigh, or shank segments. The system was selected because it was the three-dimensional motion-capture platform available in our laboratory, could be calibrated before data collection according to the manufacturer's recommended procedure, and provided a sampling frequency of 240 Hz for recording the rapid movements associated with Jodan-tsuki. No dedicated validation or sensitivity analysis of this specific 19-marker configuration against a CAST-based or other gold-standard reference was performed as part of the present study, and no claims of validation are therefore made for this specific marker model in high-speed striking actions. The standard pre-session volumetric calibration recommended by the manufacturer was performed. As reported in the broader literature on marker-based motion analysis, estimates of segmental rotation obtained using anatomical-landmark models may be affected by soft-tissue artefact, cross-talk between rotational planes, and marker-placement error, particularly for multiplanar proximal segments such as the pelvis and hip (10, 34). These methodological limitations should be considered when interpreting the present kinematic results and are discussed further in Section 5.

### 2.5 Data processing, filtering and computation of angular velocity

Three-dimensional marker trajectories were processed using the 3DMA software package (version 2023.0; STT Systems, San Sebastián, Spain). Marker trajectories were first visually inspected for tracking errors. Small gaps, when present, were reconstructed using the interpolation procedures implemented within 3DMA. The specific interpolation algorithm and the maximum accepted gap length are proprietary software parameters that were not accessible to the investigators and therefore could not be reported.

The processing sequence was as follows: (i) raw marker-coordinate data were filtered using a fourth-order zero-lag low-pass Butterworth filter with a cut-off frequency of 10 Hz; (ii) segment orientations and joint angles were then computed from the filtered marker coordinates; and (iii) angular velocity was obtained as the first temporal derivative of the resulting angular displacement signal within the 3DMA software environment. Thus, differentiation was performed after filtering, and no additional filtering or smoothing was applied to the angular-velocity signal. The specific numerical differentiation scheme implemented within the software is proprietary and was not accessible to the investigators.

#### 2.5.1 Selection of the cut-off frequency

The 10-Hz cut-off was selected *a priori* based on filtering approaches reported for rapid striking movements recorded using optical motion-capture systems (12) and general recommendations for optical motion capture in sport applications (33). No residual analysis, frequency-content analysis, or filter-sensitivity analysis was performed for the present dataset. Because the raw, unprocessed trial-level signals were not retained, the peak angular velocity values could not be retrospectively recomputed across alternative cut-off frequencies. Consequently, the sensitivity of the reported peak angular velocity magnitudes to the selected filtering procedure could not be quantified. The reported peak values should therefore be interpreted as processing-dependent estimates obtained using the specified 10-Hz filtering procedure rather than as filter-independent measures of the underlying movement.

#### 2.5.2 Peak detection

For each analysed variable, peak angular velocity was defined as the maximum absolute angular velocity attained at any instant during the complete movement cycle, from the initial guard position to the return-to-guard position, irrespective of movement direction. Peak values were identified separately for each of the 22 retained trials and subsequently averaged across trials. The direction (sign) and timing of the individual peaks were not retained in the original analysis and could not be recovered retrospectively. Consequently, the available data do not allow determination of whether a given peak occurred during the forward-striking phase or during the recovery/retraction phase. This limitation is particularly relevant to the non-striking-side upper-limb variables, because the contralateral upper limb is actively retracted during Jodan-tsuki execution. Accordingly, the reported values should be interpreted as whole-cycle absolute peak magnitudes, rather than as phase-specific, direction-specific, or temporally sequenced peak angular velocities.

### 2.6 Definition of joint versus segment angular velocity

Because relative joint angular velocity and absolute segment angular velocity represent distinct biomechanical quantities, the angular-velocity variables reported in this study were distinguished according to the reference frame indicated by the corresponding 3DMA output.

**Relative joint angular velocity** - refers to the time derivative of angular displacement describing motion between anatomically adjacent segments. In the present manuscript, the hip, knee, ankle, elbow, and shoulder flexion–extension and abduction–adduction variables are reported as joint angular-velocity measures, consistent with the corresponding joint-motion variables provided by the 3DMA output.

Segment angular velocity relative to an external reference refers to the time derivative of segment orientation expressed relative to the laboratory/global reference frame or a specified external reference. Pelvic rotation and the shoulder variables explicitly labelled “vertical reference” in the 3DMA output are therefore reported separately from the relative joint angular-velocity variables.

Accordingly, the collective term “joint and segment angular velocity” is used throughout the manuscript where both types of variables are discussed together. Individual variables and their corresponding measurement type are identified in Table 2. Because the underlying computational definitions and rotation algorithms implemented within 3DMA are proprietary, these classifications follow the nomenclature and reference descriptions available in the software output and should be interpreted within that methodological constraint.

**Table 2:** Peak joint and segment angular velocity characteristics during right-hand jodan-tsuki execution, organised by anatomical group (*N* = 1 athlete; n_trials = 22).

Anatomical group	Segment/joint	Variable (plane of motion)	Side	Angular-velocity type and reference frame	Peak mean ± SD (°·s⁻¹)
Shared proximal segment	Pelvis	Rotation (transverse)	–	Segment; pelvis relative to the global reference frame	58.77 ± 6.20
Lower limb	Hip	Flexion–extension (sagittal)	Right	Joint; thigh relative to pelvis	33.36 ± 4.10
Lower limb	Hip	Flexion–extension (sagittal)	Left	Joint; thigh relative to pelvis	51.67 ± 6.50
Lower limb	Hip	Abduction–adduction (frontal)	Right	Joint; thigh relative to pelvis	73.78 ± 8.40
Lower limb	Hip	Abduction–adduction (frontal)	Left	Joint; thigh relative to pelvis	42.32 ± 5.30
Lower limb	Knee	Flexion–extension (sagittal)	Right	Joint; shank relative to thigh	153.97 ± 16.24
Lower limb	Knee	Flexion–extension (sagittal)	Left	Joint; shank relative to thigh	174.39 ± 18.20
Lower limb	Ankle	Dorsiflexion–plantarflexion (sagittal)	Right	Joint; foot relative to shank	237.53 ± 24.02
Lower limb	Ankle	Dorsiflexion–plantarflexion (sagittal)	Left	Joint; foot relative to shank	193.97 ± 21.10
Upper limb	Shoulder	Flexion–extension (sagittal)	Right	Joint; upper arm relative to trunk	141.40 ± 15.02
Upper limb	Shoulder	Flexion–extension (sagittal)	Left	Joint; upper arm relative to trunk	94.48 ± 9.82
Upper limb	Shoulder	Flexion–extension (sagittal)	Right	Segment; upper arm relative to the global vertical reference	137.03 ± 15.20
Upper limb	Shoulder	Flexion–extension (sagittal)	Left	Segment; upper arm relative to the global vertical reference	53.18 ± 6.21
Upper limb	Shoulder	Abduction–adduction (frontal)	Right	Joint; upper arm relative to trunk	168.45 ± 18.20
Upper limb	Shoulder	Abduction–adduction (frontal)	Left	Joint; upper arm relative to trunk	73.45 ± 8.20
Upper limb	Elbow	Flexion–extension (sagittal)	Right	Joint; forearm relative to upper arm	351.42 ± 38.12
Upper limb	Elbow	Flexion–extension (sagittal)	Left	Joint; forearm relative to upper arm	67.48 ± 7.80

*N* = 1 athlete; n_trials = 22. Peak angular velocity was defined as the maximum absolute angular velocity attained at any instant during the complete movement cycle. Peak values were identified separately within each retained trial and subsequently averaged across the 22 trials. The direction (sign) and time of occurrence of the individual peaks were not retained; therefore, the table reports whole-cycle peak magnitudes only and does not provide information regarding peak direction, phase-specific occurrence, or temporal sequencing. The shoulder variables identified as segment angular velocities relative to the global vertical reference are distinct from the relative shoulder joint angular-velocity variables. Descriptive 95% CIs around the athlete's within-session trial means and CV values are provided in Supplementary Table S8f; these should not be interpreted as population-level confidence intervals or measures of test–retest reliability.

### 2.7 Experimental procedure and non-contact execution condition

Following a standardised warm-up consisting of 10 min of light aerobic activity, dynamic stretching, and karate-specific technical drills, the athlete performed a series of right-hand Jodan-tsuki techniques under laboratory conditions.

#### 2.7.1 Non-contact execution condition

No physical target was present or struck during testing. The athlete performed each right-hand Jodan-tsuki as a non-contact technique into free space. Each trial was initiated in response to a predefined signal cue. Accordingly, the terminal portion of the forward-striking action represented a simulated terminal-extension event rather than physical impact with an external object. No impact force, impulse, or other target-contact kinetic variables were measured.

During each trial, the athlete was instructed to perform the technique with maximal speed while maintaining her usual technique. The Jodan-tsuki was executed as a coordinated whole-body action involving a forward stepping action, lower-limb movement, forward body translation, pelvic and trunk motion, shoulder motion, and terminal extension of the right upper extremity. It was therefore analysed as a whole-body movement rather than as an isolated movement of the striking arm. Approximately 30–60 s of recovery was provided between consecutive trials to minimise fatigue (35). The athlete reported no discomfort or performance limitation during testing.

#### 2.7.2 Trial selection

A total of 30 Jodan-tsuki trials were performed. All trials were reviewed for technical execution quality and data completeness before calculation and statistical analysis of the outcome variables and without reference to the resulting peak angular-velocity values. A trial was considered technically successful when the athlete completed the predefined Jodan-tsuki movement without loss of balance or interruption and when marker tracking was sufficiently complete for kinematic analysis. Eight trials did not meet these criteria and were excluded from analysis. The original trial-screening records did not retain a trial-by-trial breakdown of the specific exclusion reasons; therefore, the number of exclusions attributable to individual criteria cannot be reported retrospectively. Consequently, 22 technically successful trials (*n*ₜᵣᵢₐₗₛ = 22) were retained for analysis.

Because Jodan-tsuki was performed under a standardised non-contact laboratory condition rather than against a physical target or a moving opponent, the resulting kinematic profile should be interpreted as a laboratory-standardised execution of the technique rather than as a direct representation of in-competition performance. Previous combat-sport research indicates that task and target conditions may influence striking kinematics (36); this experimental constraint is therefore considered when interpreting the present findings and is discussed further in Section 5.

### 2.8 Movement phase definitions and descriptive temporal characterisation

For descriptive and visual characterisation of the technical structure of Jodan-tsuki, one technically successful execution was selected from the 22 retained trials and divided into eight sequential movement phases. This phase-based analysis was intended to facilitate interpretation of the technical progression of the movement and was not used to calculate trial-averaged phase durations across the 22 retained trials. The operational criteria used to identify the boundaries of the eight phases are provided in Table 3.

**Table 3:** Operational definitions of the eight sequential movement phases of right-hand jodan-tsuki (*N* = 1 athlete).

Phase	Phase name	Start event (boundary criterion)	End event (boundary criterion)
1	Guard position	First frame of the selected execution, with the athlete in a stationary fighting stance	Onset of the first visible preparatory postural adjustment
2	Loading	Onset of the first visible preparatory postural adjustment	Onset of visible forward body translation
3	Initiation	Onset of visible forward body translation	Toe-off of the rear foot
4	Pre-acceleration	Toe-off of the rear foot	Onset of visible forward displacement of the striking-side wrist marker relative to the trunk
5	Acceleration	Onset of forward displacement of the striking-side wrist marker	Peak forward velocity of the striking-side wrist marker
6	Simulated impact (terminal extension)	Peak forward velocity of the striking-side wrist marker	Maximal elbow extension of the striking limb (minimum elbow flexion angle)
7	Recovery	Maximal elbow extension of the striking limb	Onset of re-establishment of the fighting stance
8	Return-to-guard	Onset of re-establishment of the fighting stance	Re-establishment of the initial stationary fighting stance

The phase definitions were used for descriptive characterisation of one selected technically successful Jodan-tsuki execution and were not used to derive trial-averaged phase durations across the 22 retained trials. Phase boundaries were identified by visual inspection of the synchronised three-dimensional skeletal reconstruction with reference to the listed kinematic events. No automated threshold-based phase-detection algorithm was applied. Phase 6 represents the terminal extension of the non-contact striking action; no physical target contact occurred.

The phase boundaries of the selected execution were identified by visual inspection of the synchronised three-dimensional skeletal reconstruction, with reference to the identifiable kinematic events specified in Table 3. The selected execution had a total duration of 1.05 s. The duration of each phase was calculated as the difference between its start and end times and was additionally expressed as a percentage of the 1.05-s total movement duration. Thus, the phase durations and percentages reported in Table 4 describe one selected technically successful execution only and should not be interpreted as mean values, variability estimates, or temporal characteristics averaged across the 22 retained trials.

**Table 4:** Descriptive temporal characteristics of the eight sequential phases of one selected technically successful right-hand jodan-tsuki execution (total movement duration = 1.05 s).

Phase	Movement phase	Start (s)	End (s)	Duration (s)	Total movement (%)
1	Guard position	0.00	0.08	0.08	7.6
2	Loading	0.08	0.18	0.10	9.5
3	Initiation	0.18	0.28	0.10	9.5
4	Pre-acceleration	0.28	0.38	0.10	9.5
5	Acceleration	0.38	0.50	0.12	11.4
6	Simulated impact (terminal extension)	0.50	0.60	0.10	9.5
7	Recovery	0.60	0.80	0.20	19.0
8	Return-to-guard	0.80	1.05	0.25	23.8
	Total	0.00	1.05	1.05	100

*N* = 1 athlete. The values describe one selected technically successful jodan-tsuki execution used for descriptive characterisation of the movement phases and are not trial-averaged values across the 22 retained trials. Phase boundaries were not identified separately for all 22 trials; therefore, phase-specific trial-to-trial variability is not reported. Percentages may not sum exactly to 100% because of rounding.

Phase 6 is designated the simulated impact (terminal extension) phase. No physical target was struck during testing. Accordingly, this phase represents the terminal portion of the forward-striking action, defined by the transition from peak forward velocity of the striking-side wrist marker toward maximal elbow extension. The term “simulated impact” is used only to describe this kinematic portion of the non-contact technique and does not represent an actual impact event or imply measurement of impact force, impulse, or other kinetic variables.

#### 2.8.1 Descriptive temporal characterisation

For descriptive and visual characterisation of the temporal structure of Jodan-tsuki, one technically successful execution was selected from the 22 retained trials and divided into the eight sequential movement phases defined in Table 3. The selected execution had a total duration of 1.05 s. For this execution, the duration of each phase was calculated as the difference between its start and end times and was additionally expressed as a percentage of the total movement duration, allowing the temporal structure of the selected execution to be represented descriptively on a 0%–100% movement-time scale.

The phase-boundary times reported in Table 4 were derived exclusively from this single selected execution. Phase boundaries were not identified separately across all 22 retained trials; therefore, the phase durations presented in Table 4 are not trial-averaged values, and phase-specific mean ± SD, median and range, or trial-to-trial variability cannot be reported. Accordingly, Table 4 is intended solely to facilitate descriptive interpretation of the temporal organisation of the selected Jodan-tsuki execution and should not be interpreted as representing the average temporal profile of the 22 retained trials.

### 2.9 Outcome variables and operational definitions

The primary outcome measures were the peak joint and segment angular velocity variables described in Section 2.6. Secondary outcome measures included temporal–spatial characteristics, joint range of motion (ROM), and task-related bilateral differences.

#### 2.9.1 Kinematic variables

Trunk tilt, trunk lateral flexion, cervical flexion–extension, cervical lateral flexion, cervical rotation, shoulder flexion–extension, shoulder abduction–adduction, elbow flexion–extension, hip flexion–extension, hip abduction–adduction, knee flexion–extension, and ankle dorsiflexion–plantarflexion were analysed. For each variable, the minimum angle, maximum angle, and ROM were calculated. ROM was calculated within each individual trial as the difference between the maximum and minimum angle attained during that trial, and the trial-specific ROM values were subsequently averaged across the 22 retained trials. Thus, ROM was not calculated by subtracting the across-trial mean minimum angle from the across-trial mean maximum angle. These trunk, cervical, and limb kinematic variables were selected because joint ROM during technique execution has previously been examined in relation to skill level in karate striking (4, 37).

#### 2.9.2 Temporal–spatial variables

Temporal–spatial variables associated with the stepping action accompanying Jodan-tsuki were obtained from the temporal–spatial output of the 3DMA software. The software-reported variables included cadence, right and left flight time, total flight time, support time, step length, centre-of-gravity (COG) measures, braking/propulsion distance, and support distance. Where the exact underlying computational algorithm was not accessible to the investigators, the variable is reported according to the terminology provided by the 3DMA software rather than being assigned a retrospectively inferred computational definition.

**Cadence (steps·min^−1^)** - was reported by the 3DMA temporal–spatial module. Because the present task comprised a single stepping action rather than continuous locomotion, cadence is interpreted only as a software-derived descriptor of the rapidity of the stepping action and not as a sustained stepping frequency.

**Right/left flight time (s)** - represent the software-reported temporal measures associated with the non-contact interval of the respective limb during the stepping action. Total flight time (s) is reported separately according to the corresponding 3DMA output.

**Support time (s)** - the duration during which the respective foot remained in contact with the floor during the stepping action.

**Step length (mm)** - represents the software-reported spatial measure of the stepping action. Because the available study records do not permit definitive assignment of the two horizontal global axes to participant-relative anteroposterior and mediolateral directions, step length is described according to its biomechanical meaning rather than assigned to an unverified global axis.

**Centre of gravity (COG)** - is the terminology used by the 3DMA software for the model-derived whole-body spatial quantity reported in the temporal–spatial output. The underlying segmental anthropometric and inertial computations were not accessible to the investigators; therefore, no additional computational definition is inferred. The term “COG” is retained throughout the manuscript for consistency with the software output.

**COG vertical oscillation (mm)** - represents the software-reported vertical excursion of the estimated COG during the analysed movement. In accordance with the laboratory coordinate convention described in Section 2.3, the vertical direction corresponds to the global *Y*-axis.

**Braking/propulsion distance (mm)** - are reported according to the corresponding spatial variables generated by the 3DMA temporal–spatial module. Because the exact software-specific computational definitions and sign conventions for these measures were not available to the investigators, no additional anatomical or axis-based interpretation is imposed retrospectively. These variables are therefore interpreted descriptively within the nomenclature provided by 3DMA.

#### 2.9.3 Rationale for the temporal–spatial variables

Jodan-tsuki is a whole-body striking action that may incorporate a forward stepping or lunging component together with the upper-limb strike. Accordingly, temporal–spatial variables were included to characterise the stepping component accompanying the Jodan-tsuki execution and to complement the joint- and segment-level kinematic analysis. Previous karate research has indicated that movement and stepping conditions can influence the kinematic characteristics of punching actions (38). In the present study, cadence, flight time, and support time were used as descriptive temporal characteristics of the stepping action, whereas step length, braking/propulsion distance, and support distance were used as descriptive spatial characteristics. COG vertical oscillation was included to characterise the vertical displacement of the model-derived COG during the movement (39). Collectively, these variables were interpreted as descriptive features of the whole-body movement strategy rather than as direct measures of mechanical efficiency, propulsive force, or striking effectiveness.

Hand/wrist measurement. Markers 8 and 9 (Table 1) were positioned on the wrist region of the distal forearm/hand segment and not on the striking fist. The forward velocity of marker 9 (right wrist region) was used only as a kinematic event signal to identify the transition between the acceleration and simulated impact/terminal-extension phases (Table 3). Specifically, the instant of peak forward velocity of the right wrist-region marker defined the end of Phase 5 and the beginning of Phase 6. The magnitude of this marker velocity was not analysed or reported as a performance outcome.

Because the marker was positioned at the wrist region rather than on the fist, its linear velocity should not be interpreted as fist velocity. No dedicated marker was placed on the striking fist, and wrist-joint-centre velocity, hand-segment linear velocity, and fist linear velocity were not computed as outcome variables in the present study. Accordingly, the principal velocity outcomes reported in this manuscript are joint and segment angular velocity measures, as defined in Section 2.6.

### 2.10 Quantification of task-related bilateral differences

Bilateral differences were quantified using the Asymmetry Index (AI): AI (%) = |Right−Left|/[0.5 × (Right + Left)] × 100, following the methodological recommendations of Bishop et al. (17). The AI was calculated from the trial-averaged right and left means reported in Tables 5, 6 (i.e., a single AI value per variable), and not as a paired, trial-by-trial calculation subsequently averaged across trials. Consequently, no standard deviation, confidence interval or other variability estimate can be reported for the AI values, and the within-trial pairing of right and left values is not preserved. Future studies should calculate AI on a paired trial-by-trial basis and report its mean and dispersion.

**Table 5:** Bilateral upper-limb range-of-motion characteristics during right-hand jodan-tsuki (*N* = 1 athlete; n_trials = 22).

Variable	Left mean ± SD (°)	Right mean ± SD (°)	AI (%)
Shoulder flexion ROM	19 ± 2.3	62 ± 6.7	106.2
Shoulder abduction ROM	12 ± 1.4	11 ± 1.4	8.7
Elbow flexion ROM	36 ± 4.2	14 ± 1.6	88.0

*N* = 1 athlete; n_trials = 22. ROM = range of motion, computed within each trial and then averaged across trials; AI = asymmetry index, calculated from the trial-averaged right and left means shown (a single value per variable, without a variability estimate; Section 2.10). AI values express task-related bilateral differences during a unilateral right-hand technique and are not indices of functional deficit. Full 95% confidence intervals, describing the uncertainty of this athlete's within-session trial mean, are reported in Supplementary Table S8c.

**Table 6:** Bilateral lower-limb range-of-motion characteristics during right-hand jodan-tsuki (*N* = 1 athlete; n_trials = 22).

Variable	Left mean ± SD (°)	Right mean ± SD (°)	AI (%)
Hip flexion ROM	80 ± 8.4	35 ± 4.2	78.3
Hip abduction ROM	41 ± 5.2	25 ± 3.2	48.5
Knee flexion ROM	43 ± 4.8	39 ± 4.2	9.8
Ankle dorsiflexion–plantarflexion ROM	52 ± 5.9	47 ± 5.4	10.1

*N* = 1 athlete; n_trials = 22. ROM computed within each trial and then averaged; AI calculated from the trial-averaged right and left means shown (Section 2.10). Full 95% confidence intervals are reported in Supplementary Table S8d.

Because the striking (right) and non-striking/supporting (left) limbs perform mechanically non-equivalent roles during a unilateral punching action, all AI values reported here are interpreted as task-related bilateral differences during right-hand Jodan-tsuki and not as evidence of a functional deficit, maladaptation or generalised limb asymmetry. No validated interpretive thresholds exist for the kinematic variables analysed here; therefore the qualitative descriptors “low”, “moderate”, “high” and “symmetrical” have been removed from the revised manuscript, and the bilateral differences are described numerically and relative to one another within the present dataset only.

### 2.11 Statistical analysis

All statistical analyses were performed using IBM SPSS Statistics (version 29.0; IBM Corp., Armonk, NY, USA). Given the descriptive case-study design (*N* = 1 athlete; n_trials = 22 retained trials), the analyses were intended to characterise the within-athlete, within-session biomechanical features of Jodan-tsuki rather than to support population-level statistical inference. For variables quantified across the 22 retained trials, descriptive statistics are presented as mean ± standard deviation (SD).

#### 2.11.1 Confidence intervals

The 95% confidence intervals (95% CI) reported in Tables 1, 5–7 and in Supplementary Tables S8a–S8f were calculated as mean ± t₀.₉₇₅,₍₂₁₎ × SD/√22, where t₀.₉₇₅,₍₂₁₎ = 2.080 is the two-tailed critical value of Student's t distribution with 21 degrees of freedom. These intervals quantify the uncertainty around this athlete's within-session trial mean and are not population-level confidence intervals; they describe how precisely the mean of the 22 repeated trials of this one athlete was estimated, and permit no inference about elite karate athletes as a population. This interpretation is restated in the note of every table in which the intervals appear.

**Table 7:** Trunk and cervical kinematic characteristics during right-hand jodan-tsuki (*N* = 1 athlete; n_trials = 22).

Segment	Variable	Minimum mean ± SD (°)	95% CI lower	95% CI upper	Maximum mean ± SD (°)	95% CI lower	95% CI upper
Trunk	Tilt	−6 ± 0.5	−6.22	−5.78	1 ± 0.01	0.996	1.004
Trunk	Lateral flexion	−5 ± 0.4	−5.18	−4.82	8 ± 0.9	7.60	8.40
Neck	Flexion–extension	−25 ± 2.8	−26.24	−23.76	−3 ± 0.41	−3.18	−2.82
Neck	Lateral flexion	−29 ± 3.2	−30.42	−27.58	10 ± 1.3	9.42	10.58
Neck	Rotation	1 ± 0.01	0.996	1.004	30 ± 4.2	28.14	31.86

*N* = 1 athlete; n_trials = 22. Values are mean ± SD across the 22 retained trials. The 95% CIs were calculated as mean ± *t*₀.₉₇₅,₂₁ × (SD/√22), where *t*₀.₉₇₅,₂₁ = 2.080. In this *N* = 1 repeated-trial case study, these intervals are reported descriptively around the athlete's within-session trial mean and should not be interpreted as population-level confidence intervals. Signed angular values are reported according to the directional conventions of the corresponding 3DMA output variables, as described in Section 2.4.

#### 2.11.2 Within-session variability

Coefficients of variation (CV%) were calculated as (SD/|mean|) × 100 to describe relative trial-to-trial variability across the 22 retained trials within the single testing session. Because the study did not employ a repeated-session or test–retest design and marker placement was maintained throughout a single testing session, the CV values are interpreted solely as descriptive measures of within-session trial-to-trial variability. They should not be interpreted as estimates of test–retest reliability, measurement reproducibility, or between-session stability. Accordingly, the terms “reliable”, “reliability”, and “reproducible” were not used to characterise the CV values in the revised manuscript.

No formal population-level inferential tests or multivariate statistical models were applied. Reliability statistics such as the intraclass correlation coefficient (ICC) and standard error of measurement (SEM) were also not calculated because the study was not designed as a test–retest reliability investigation. The 22 retained trials represent repeated observations from a single athlete rather than independent observations from a sample of athletes and therefore were used only for within-athlete descriptive characterisation.

## 3 Results

The principal observations of the present investigation were: (i) distinct peak joint and segment angular velocity magnitudes across the pelvis, lower extremity, and upper extremity, with the largest magnitude observed at the striking-side elbow; (ii) task-related bilateral differences of varying magnitude across the shoulder, elbow, hip, knee, and ankle variables; and (iii) comparatively small trunk angular excursions alongside larger cervical excursions. The temporal order of the segmental angular-velocity peaks was not quantified and is therefore not reported.

### 3.1 Sequential movement phases of right-hand jodan-tsuki

The sequential structure of the selected right-hand Jodan-tsuki execution is illustrated in Figure 2. For descriptive characterisation, the movement was divided into eight consecutive phases: (1) guard position, (2) loading, (3) initiation, (4) pre-acceleration, (5) acceleration, (6) simulated impact (terminal extension), (7) recovery, and (8) return-to-guard. The operational boundary criteria for these phases are provided in Table 3, and the corresponding temporal characteristics of the selected execution are presented in Table 4. The phase decomposition was used to facilitate descriptive interpretation of the technical movement sequence and was not intended as a trial-averaged temporal analysis of the 22 retained trials.

**Figure 2:**
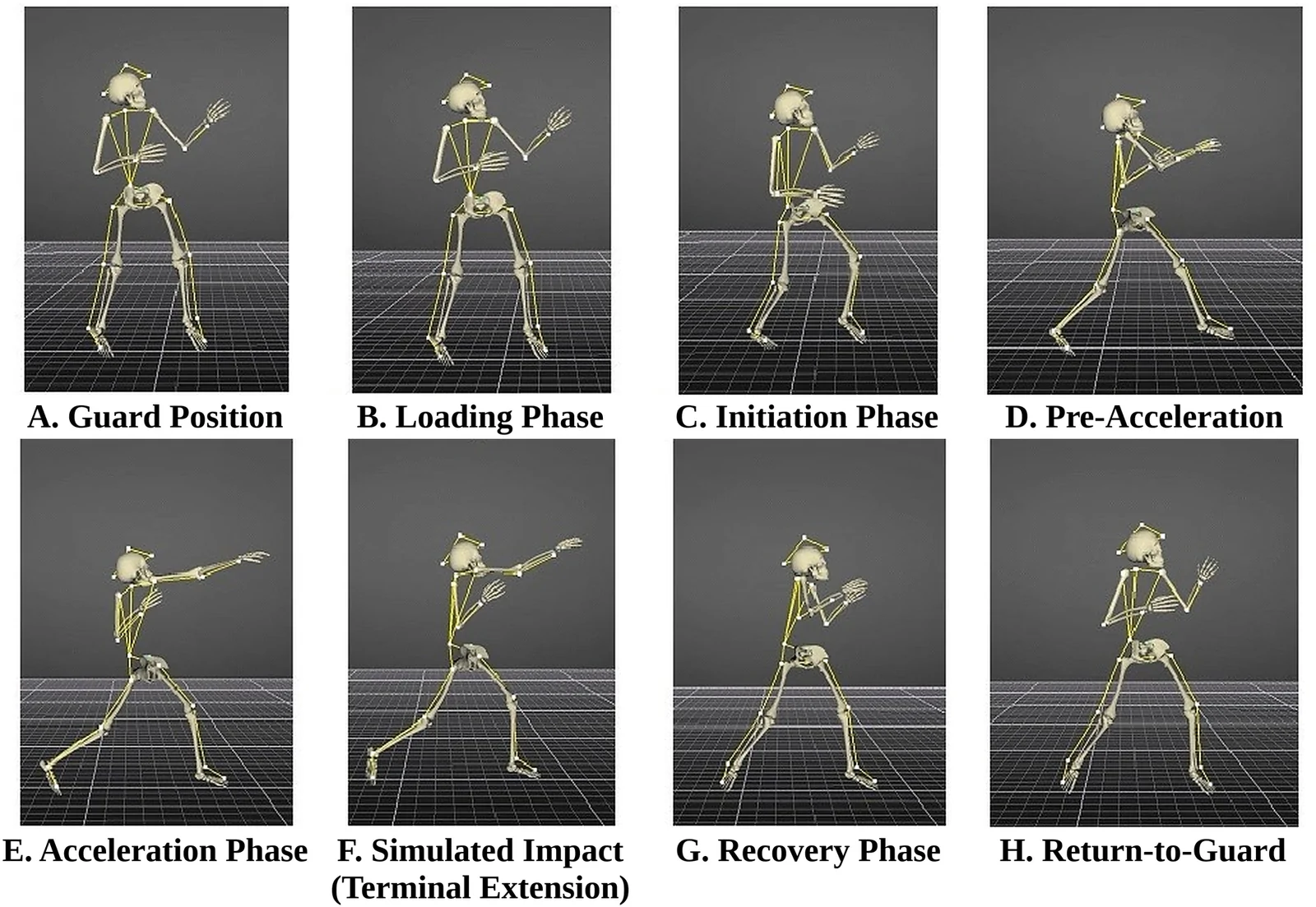
Sequential movement phases of one selected technically successful right-hand Jodan-tsuki execution: **(A)** Guard Position, **(B)** Loading, **(C)** Initiation, **(D)** Pre-Acceleration, **(E)** Acceleration, **(F)** Simulated Impact (Terminal Extension), **(G)** Recovery, and **(H)** Return-to-Guard. The sequence is provided for descriptive visual characterization of one selected execution and does not represent trial-averaged phase timing across the 22 retained trials. No physical target was present or struck during testing.

In this selected technically successful execution, the guard phase accounted for 7.6% of the total movement duration; the loading, initiation, and pre-acceleration phases each represented approximately 9.5%; the acceleration phase lasted 0.12 s (11.4%), followed by the simulated impact (terminal-extension) phase lasting 0.10 s (9.5%); and the recovery and return-to-guard phases accounted for the largest proportions of the movement cycle, lasting 0.20 s (19.0%) and 0.25 s (23.8%), respectively.

Figure 2 provides a descriptive visualisation of the whole-body configurations observed across the eight defined phases of the selected Jodan-tsuki execution. The illustrated sequence is intended to facilitate interpretation of the technical structure of the movement and should not be interpreted as a quantitative analysis of intersegmental coordination or temporal sequencing. No formal coordination metric, such as continuous relative phase or vector coding, and no analysis of the temporal order of segmental angular-velocity peaks was performed.

Supplementary Figures S2–S7 present temporal joint-kinematic waveforms obtained from one selected technically successful Jodan-tsuki execution among the 22 retained trials. The selected execution was used to provide qualitative visualisation of the time-varying kinematic patterns during the technique and was not selected on the basis of its peak angular-velocity values. These waveforms represent a single execution and should not be interpreted as ensemble-averaged profiles across the 22 retained trials. All quantitative descriptive statistics reported in the main results tables and supplementary statistical tables were calculated from the complete set of 22 technically successful trials, except where explicitly identified as descriptive data from the single selected execution.

### 3.2 Temporal and spatial characteristics

The temporal–spatial characteristics of right-hand Jodan-tsuki execution across the 22 retained trials are presented in Table 8. The operational definition of each variable is given in Section 2.9 and must be consulted when interpreting these values, because several of them are software-derived outputs of the 3DMA temporal–spatial module rather than independently computed quantities.

**Table 8:** Temporal–spatial characteristics of right-hand jodan-tsuki (*N* = 1 athlete; n_trials = 22).

Variable	Mean ± SD
Cadence (steps·min⁻¹) ᵃ	222.22 ± 23.50
Flight time, total (s) ᵇ	0.39 ± 0.04
Support time (s)	0.24 ± 0.03
Step length (mm)	171.03 ± 20.10
Right flight time (s)	0.199 ± 0.020
Left flight time (s)	0.199 ± 0.021
COG vertical oscillation (mm)	156.43 ± 15.40
Right braking distance (mm) ᶜ	−55.81 ± 6.41
Left braking distance (mm) ᶜ	−11.70 ± 1.81
Right propulsion distance (mm) ᶜ	−47.84 ± 5.41
Left propulsion distance (mm) ᶜ	−9.78 ± 1.40
Right support distance (mm) ᵈ	609.39 ± 75.40
Left support distance (mm) ᵈ	753.08 ± 82.41

*N* = 1 athlete; n_trials = 22. Values are mean ± SD across the 22 retained trials. COG = centre of gravity, following the terminology used in the 3DMA software output. Full 95% confidence intervals and coefficients of variation (CV%) for these variables are provided in Supplementary Table S8a. The 95% CIs describe the distribution of repeated within-session observations around this athlete's trial mean and should not be interpreted as population-level confidence intervals. Operational considerations for the software-derived temporal–spatial variables are provided in Section 2.9.

The athlete demonstrated a mean step length of 171.03 ± 20.10 mm and a mean COG vertical oscillation of 156.43 ± 15.40 mm across the 22 retained trials. Mean right and left flight times were numerically similar (0.199 ± 0.020 s and 0.199 ± 0.021 s, respectively). Side-to-side differences were observed in the braking, propulsion, and support-distance variables. The absolute magnitudes of the right braking and propulsion distances were greater than the corresponding left-side values, whereas the left support distance was greater than the right support distance. These observations are reported descriptively and are not interpreted as evidence of limb-specific mechanical efficiency or functional asymmetry.

Across the temporal–spatial variables, CV values ranged from 9.84% to 15.47%. These values describe the relative trial-to-trial variability observed across the 22 retained trials within the single testing session. They should not be interpreted as estimates of test–retest reliability, measurement reproducibility, or between-session stability.

### 3.3 Trunk and cervical kinematic characteristics

The trunk and cervical kinematic characteristics observed across the 22 retained right-hand Jodan-tsuki trials are presented in Table 7. The trunk variables showed comparatively small angular ranges, with mean minimum and maximum values of −6 ± 0.5° and 1 ± 0.01°, respectively, for trunk tilt, and −5 ± 0.4° and 8 ± 0.9°, respectively, for trunk lateral flexion. Larger angular ranges were observed for the cervical variables, particularly lateral flexion and rotation.

Larger angular ranges were observed for the cervical variables than for the trunk variables. For cervical flexion–extension, the mean minimum and maximum values were −25 ± 2.8° and −3 ± 0.41°, respectively, while cervical lateral flexion showed corresponding values of −29 ± 3.2° and 10 ± 1.3°. Cervical rotation showed mean minimum and maximum values of 1 ± 0.01° and 30 ± 4.2°, respectively. The CV values for these variables describe the relative trial-to-trial variability observed across the 22 retained trials within the single testing session and should not be interpreted as measures of test–retest reliability or reproducibility.

The differences between the trunk and cervical angular ranges are reported as descriptive observations for this athlete. No inferential comparison between the trunk and cervical variables was performed. Accordingly, these findings should not be interpreted as demonstrating a general difference in trunk and cervical mobility during Jodan-tsuki beyond the participant studied. Temporal waveforms of trunk tilt and trunk lateral flexion from one selected technically successful execution are presented in Supplementary Figure S2, with additional cervical kinematic waveforms from the same selected execution presented in Supplementary Figure S3. These waveforms are provided for qualitative visualisation and do not represent ensemble-averaged profiles across the 22 retained trials.

### 3.4 Bilateral upper-limb range-of-motion characteristics

The bilateral upper-limb ROM characteristics during right-hand Jodan-tsuki execution are presented in Table 5. Bilateral shoulder and elbow kinematic waveforms throughout the movement cycle are provided in Supplementary Figures S4, S5.

The right shoulder exhibited a greater flexion ROM (62 ± 6.7°) than the left shoulder (19 ± 2.3°), corresponding to the largest bilateral difference observed among the upper-limb variables (AI = 106.2%). Shoulder abduction ROM differed little between limbs (left 12 ± 1.4°; right 11 ± 1.4°; AI = 8.7%). At the elbow, the left limb showed a greater flexion ROM (36 ± 4.2°) than the right limb (14 ± 1.6°; AI = 88.0%), a pattern consistent with the mechanically different roles of the striking and the retracting/guarding limb. Vences Brito et al. (4) reported that dominant- and non-dominant-side joints follow different temporal contribution patterns during karate punching, and a recent study of elite boxers similarly described complementary roles for the two limbs during straight punches (40).

### 3.5 Bilateral lower-limb range-of-motion characteristics

The largest inter-limb difference in the lower extremity was identified in hip flexion ROM, with a greater value on the left (80 ± 8.4°) than on the right (35 ± 4.2°; AI = 78.3%). Hip abduction ROM was also greater on the left (41 ± 5.2°) than on the right (25 ± 3.2°; AI = 48.5%). Knee and ankle ROM differed less between limbs: knee flexion ROM was 43 ± 4.8° (left) and 39 ± 4.2° (right; AI = 9.8%), and ankle dorsiflexion–plantarflexion ROM was 52 ± 5.9° (left) and 47 ± 5.4° (right; AI = 10.1%). Bilateral hip, knee and ankle kinematic waveforms are presented in Supplementary Figures S6, S7.

### 3.6 Summary of task-related bilateral differences

A complete summary of the task-related bilateral differences is provided in Supplementary Table S8e. Among the variables examined, the largest numerical AI values were observed for shoulder flexion (106.2%), elbow flexion (88.0%), hip flexion (78.3%), and hip abduction (48.5%), whereas smaller numerical AI values were observed for shoulder abduction (8.7%), knee flexion (9.8%), and ankle dorsiflexion–plantarflexion (10.1%). These values are reported numerically and are not assigned qualitative classifications because no validated interpretive thresholds were applied to the kinematic variables examined in the present study.

Within this athlete and this specific right-hand Jodan-tsuki task, the numerical bilateral differences were greater for several shoulder, elbow, and hip variables than for the knee and ankle variables. This pattern is reported descriptively and should not be interpreted as evidence of functional deficit, pathological asymmetry, or a general proximal-to-distal asymmetry pattern.

### 3.7 Peak joint and segment angular velocity characteristics

The peak joint and segment angular velocity characteristics observed across the 22 retained right-hand Jodan-tsuki trials are presented in Table 2. The variables are organised into anatomically defined lower-limb and upper-limb groups, with the pelvis presented separately as a shared proximal segment. Each variable is identified according to whether it represents relative joint angular velocity or segment angular velocity relative to an external reference, as defined in Section 2.6. The reported values represent whole-cycle absolute peak magnitudes; the direction and temporal order of the individual peaks were not quantified.

The largest mean peak angular velocity magnitude among the analysed variables was observed for right elbow flexion–extension (351.42 ± 38.12°·s⁻¹). Among the lower-limb variables, the right ankle showed the largest mean peak magnitude (237.53 ± 24.02°·s⁻¹), followed numerically by the left ankle (193.97 ± 21.10°·s⁻¹), left knee (174.39 ± 18.20°·s⁻¹), and right knee (153.97 ± 16.24°·s⁻¹). Within the upper limb, right shoulder abduction–adduction showed a mean peak magnitude of 168.45 ± 18.20°·s⁻¹, while right shoulder joint flexion–extension reached 141.40 ± 15.02°·s⁻¹. Pelvic rotation showed a mean peak magnitude of 58.77 ± 6.20°·s⁻¹.

#### 3.7.1 Distribution of peak magnitudes across anatomical groups

Within the striking-side upper limb, the mean peak magnitudes were 58.77°·s⁻¹ for pelvic rotation, 141.40°·s⁻¹ for right shoulder joint flexion–extension, 168.45°·s⁻¹ for right shoulder joint abduction–adduction, and 351.42°·s⁻¹ for right elbow flexion–extension. Within the lower limb, no uniform ordering of peak magnitudes was observed across all variables. The right and left hip flexion–extension values (33.36 and 51.67°·s⁻¹, respectively) were lower than the pelvic rotation value (58.77°·s⁻¹), whereas right hip abduction–adduction showed a value of 73.78°·s⁻¹. The knee values ranged from 153.97 to 174.39°·s⁻¹ and the ankle values from 193.97 to 237.53°·s⁻¹. Because the analysed joint and segment variables represent different biomechanical quantities and their peak timing was not retained, these magnitude differences should not be interpreted as evidence of a single monotonic proximal-to-distal temporal sequence.

Side-to-side differences in peak angular velocity magnitude were also observed for several variables. Right elbow flexion–extension showed a mean peak magnitude of 351.42 ± 38.12°·s⁻¹ compared with 67.48 ± 7.80°·s⁻¹ on the left. The right-side shoulder variables also showed numerically greater peak magnitudes than their corresponding left-side variables, whereas side-to-side differences of varying magnitude were observed at the hip, knee, and ankle. Because the reported peaks represent maximum absolute values over the complete movement cycle and their direction and timing were not retained, it cannot be determined whether individual peaks—particularly those of the non-striking upper limb-occurred during the forward-striking or recovery/retraction portion of the movement.

Across the peak joint and segment angular velocity variables, CV values ranged from 10.11% to 12.58%. These values describe the relative trial-to-trial variability observed across the 22 retained trials within the single testing session and should not be interpreted as estimates of test–retest reliability, measurement reproducibility, or between-session stability.

## 4 Discussion

The purpose of this descriptive case study was to characterise peak joint and segment angular velocity magnitudes, joint range of motion, trunk and cervical kinematics, temporal–spatial characteristics, and task-related bilateral differences during right-hand Jodan-tsuki in a world champion female karate athlete. The findings are interpreted as within-athlete descriptive observations from a single testing session and are not intended to support population-level generalisation.

### 4.1 Distribution of peak angular velocity magnitudes

The largest mean peak angular velocity magnitude among the analysed variables was observed for striking-side elbow flexion–extension (351.42°·s⁻¹). Lower mean peak magnitudes were observed for pelvic rotation (58.77°·s⁻¹), the hip variables (33.36–73.78°·s⁻¹), knee flexion–extension (153.97–174.39°·s⁻¹), ankle dorsiflexion–plantarflexion (193.97–237.53°·s⁻¹), and the shoulder variables (53.18–168.45°·s⁻¹). Within the striking-side upper limb, the numerical peak magnitudes were greater at the shoulder than at the pelvis and greatest at the elbow. Within the lower limb, the knee and ankle peak magnitudes were numerically greater than the hip values. However, the analysed variables did not form a single monotonic proximal-to-distal magnitude gradient across the body: the hip flexion–extension values were lower than the pelvic rotation value, and several shoulder values were lower than the knee and ankle values. Accordingly, these findings are best interpreted as a distribution of whole-cycle peak angular velocity magnitudes across different joint and segment variables rather than as evidence of a uniform proximal-to-distal sequence.

This distinction is important because proximal-to-distal sequencing refers to the temporal ordering of segmental or joint kinematic events during movement (11). In the present study, the reported values represent maximum absolute angular velocity magnitudes identified over the complete movement cycle. The direction and timing of the individual peaks were not retained, and no phase-lag, continuous relative phase, vector-coding, or other kinematic-sequence timing analysis was performed. Consequently, the present data cannot demonstrate the temporal order of the angular-velocity peaks or establish a proximal-to-distal sequencing strategy. They also cannot demonstrate intersegmental momentum transfer, because segmental momentum and its transfer were not directly quantified. Although proximal-to-distal sequencing has been described as an important organisational principle in explosive multisegmental actions (11), the present findings should be interpreted only in terms of the observed peak-magnitude distribution.

The striking-side elbow showed the greatest peak angular velocity magnitude among the variables examined in this athlete. This observation is consistent with previous descriptions of rapid upper-limb motion during karate punching (2, 4, 6, 39). However, the present kinematic data alone do not establish that the elbow was the primary determinant of striking performance or that the observed elbow angular velocity resulted from momentum transferred from proximal segments. Ground reaction forces, joint moments, mechanical power, impact force, and muscle activation were not measured; therefore, kinetic, neuromuscular, or causal interpretations cannot be derived from the present data. Similarly, the comparatively large knee and ankle peak angular velocity magnitudes indicate rapid lower-limb angular motion during the analysed technique but should not be interpreted as direct measures of lower-limb force, power, or mechanical contribution to striking performance (38, 41).

### 4.2 Task-related bilateral differences

Bilateral differences were most pronounced in shoulder flexion ROM (AI = 106.2%), elbow flexion ROM (AI = 88.0%) and hip flexion ROM (AI = 78.3%), and smallest in knee flexion (AI = 9.8%), ankle dorsiflexion–plantarflexion (AI = 10.1%) and shoulder abduction (AI = 8.7%). These values must be interpreted with an important caveat: during a unilateral striking task the striking (right) and the supporting/guarding (left) limbs perform mechanically non-equivalent roles. A high AI value at a given joint may therefore reflect the structure of the task itself as much as any neuromuscular adaptation, and the two cannot be separated in the present design. Accordingly, the AI values are interpreted throughout as task-related bilateral differences during right-hand Jodan-tsuki, and not as evidence of a functional deficit, a maladaptation or a generalised limb asymmetry. Consistent with this interpretation, the knee and ankle - which perform more comparable roles across limbs during the stepping-striking action - showed the smallest differences. Genuine motor-strategy asymmetry also cannot be fully distinguished from differences associated with the standardised single-sided stance, the sensitivity of the marker configuration to multiplanar rotation (Section 2.4), or software-based reconstruction. Comparable task-related bilateral differences have been described in karate, boxing and other unilateral striking disciplines (4, 12, 18, 40). A study including both right- and left-hand punching conditions would be required to separate task-role effects from more general functional asymmetry.

### 4.3 Trunk and cervical kinematics

Trunk angular excursions were smaller than cervical excursions, with the largest cervical value observed for axial rotation. This pattern is reported descriptively and should not be interpreted as evidence of trunk stability, gaze control, or visual tracking because these variables were not directly measured. Specifically, no eye-tracking, centre-of-pressure, or perturbation data were collected. Accordingly, the present findings do not establish whether the observed trunk and cervical kinematics contributed to postural stability or visual orientation. Previous literature on visual–motor coordination and gaze behaviour in combat sports (2, 42, 43) provides broader contextual evidence regarding visual and postural demands during dynamic combat-sport actions but does not provide a direct quantitative benchmark for the trunk and cervical angular ranges observed during the present non-contact Jodan-tsuki execution.

### 4.4 Temporal–spatial characteristics and trial-to-trial consistency

The temporal–spatial data describe a rapid stepping action with a short support time and a measurable forward translation of the estimated centre of gravity. Because directly comparable reference values for stepping Jodan-tsuki in elite karate athletes are limited, these values are presented descriptively rather than against a population-based benchmark. In addition, several temporal–spatial variables were software-derived outputs for which the internal computational procedures were not accessible, as described in Section 2.9. CV values between approximately 10% and 15% describe the relative trial-to-trial variability observed across the retained trials within the present testing session. Because no test–retest design was used and the markers were applied only once, these values should not be interpreted as estimates of measurement reliability, between-session reproducibility, or motor-control quality.

### 4.5 Interpretive boundaries of the laboratory condition

The technique was performed as a non-contact action into free space under laboratory conditions, with no physical target present or struck, rather than against a physical target or an opponent. Previous research indicates that striking kinematics may differ according to task and target conditions (36). Therefore, the kinematic characteristics observed in the present study should be interpreted specifically within the standardized non-contact laboratory condition used here and should not be assumed to represent this athlete's in-competition execution. Future studies should examine Jodan-tsuki under more ecologically valid conditions, including physical-target and opponent-based tasks.

## 5 Limitations and future directions

### 5.1 Single-athlete design

The findings derive from one world-champion female karate athlete of a specific age and weight category and cannot be generalised to the broader karate population, including male athletes and athletes of different age groups, weight categories, competitive levels or technical backgrounds. This applies even within the elite population.

### 5.2 Kinematic measurements only

Ground reaction forces, joint moments, mechanical power, impact force and electromyographic activity were not recorded. In addition, peak linear hand/fist velocity could not be calculated because the 19-marker configuration contained no dedicated marker on the striking fist. Consequently the present findings cannot establish the magnitude or temporal characteristics of force, power, muscle activation or momentum transfer.

### 5.3 Temporal sequencing not quantified

The direction and the time of occurrence of each angular velocity peak were not retained, and no phase-lag, continuous relative phase, vector-coding or kinematic-sequence timing analysis was performed. The present data therefore describe peak magnitudes only and cannot confirm proximal-to-distal sequencing or intersegmental coordination. Because the peaks were identified as absolute maxima over the complete movement cycle, it also cannot be determined whether a given peak - particularly on the non-striking side - occurred during the forward-striking action or during retraction.

### 5.4 Biomechanical model

The 19-marker anatomical-landmark model lacked independent technical clusters and was not validated against a CAST/ISB-compliant reference model, leaving it susceptible to soft-tissue artefact and cross-talk, particularly for multiplanar proximal segments such as the pelvis and hip. Furthermore, the joint-centre definitions, local coordinate system definitions, Cardan rotation sequences, numerical differentiation scheme and interpolation algorithm are proprietary to the 3DMA software and were not accessible to the investigators. Independent reproduction of the present processing pipeline by other laboratories is therefore not fully possible, and some reported joint-motion magnitudes and bilateral difference values may partly reflect measurement-model characteristics rather than purely physiological differences. This limitation applies with particular force to the pelvis, hip, shoulder and cervical variables.

### 5.5 Signal processing not verified by sensitivity analysis

The 10-Hz cut-off was chosen *a priori*; no residual analysis, frequency-content analysis or filter sensitivity analysis was performed, and the raw trial-level signals are no longer available for such an analysis. The robustness of the reported peak magnitudes to the selected processing procedure therefore remains unverified.

### 5.6 Phase identification

Movement phases were identified by visual inspection of the three-dimensional skeletal reconstruction rather than by an automated threshold-based algorithm, and the phase-boundary times reported in Table 4 derive from one selected technically successful execution. Phase-specific trial-to-trial variability was therefore not quantified. Accordingly, the phase durations reported in Table 4 should be interpreted as descriptive characteristics of the selected execution rather than as average temporal characteristics across all 22 retained trials.

### 5.7 Asymmetry index computation

The AI values were calculated from the trial-averaged right and left means rather than from paired trial-level values; therefore, within-trial pairing was not preserved, and no variability estimate for the AI could be reported. Future studies should calculate AI on a paired trial-by-trial basis and report its mean and dispersion.

### 5.8 Representative waveforms

The temporal joint-kinematic waveforms presented in Supplementary Figures S2–S7 were obtained from one selected technically successful execution and are provided as qualitative illustrations; ensemble-averaged waveforms with variability bands across all 22 retained trials could not be produced.

### 5.9 Laboratory, non-contact condition

The technique was executed into free space under laboratory conditions, with no physical target present or struck, rather than against a physical target or moving opponent. Although this standardised non-contact condition facilitated consistency across trials, it limits the ecological validity of the findings (36).

### 5.10 Dominance definition

The right side was designated as the preferred, task-defined striking side rather than as formally assessed limb dominance because no standardised handedness or limb-dominance assessment was performed.

Future studies should include larger and more diverse samples across sex, age, weight category and competitive level, and should combine three-dimensional motion capture with electromyography, kinetic measurements, validated marker models, automated phase-detection procedures, retained peak-timing data and trial-normalised ensemble waveform analyses under more ecologically valid conditions. Such approaches would permit a direct examination of intersegmental coordination, kinetic-chain dynamics and the generalisability of the characteristics observed here.

## 6 Practical considerations

The following considerations follow from the descriptive nature of this single-athlete case study and should not be read as normative thresholds or as causal training prescriptions; none of them has been tested experimentally in the present work.

The angular velocity values recorded at both lower- and upper-extremity joints are consistent with Jodan-tsuki being executed as a whole-body action rather than as an isolated upper-limb movement in this athlete, which may be relevant when coaches consider how the technique is taught and analysed.

Numerically greater task-related bilateral differences were observed at several shoulder, elbow, and hip variables than at the knee and ankle variables in this athlete. Practitioners who monitor inter-limb differences during unilateral striking techniques should be aware that such values partly reflect the mechanically distinct roles of the striking and supporting limbs and should not be interpreted directly as indicators of functional imbalance.

The values reported here may serve as a descriptive point of comparison for future single- and multi-athlete biomechanical investigations of elite karate athletes. They are not normative reference values and should not be used as performance benchmarks or selection criteria.

## 7 Conclusions

This study provides a detailed three-dimensional biomechanical description of right-hand Jodan-tsuki execution in one world champion female karate athlete (*N* = 1 athlete; n_trials = 22). In this athlete, the technique was characterised by the highest peak angular velocity at the striking-side elbow (351.42 ± 38.12°·s⁻¹), measurable peak angular velocities at the ankle, knee, and shoulder, and greater task-related bilateral differences at selected shoulder, elbow, and hip variables alongside smaller numerical differences at the knee and ankle. Trunk angular excursions were smaller than cervical excursions.

Peak angular velocity magnitudes differed across the analysed joint and segment variables. Because peak timing was not quantified, these data do not demonstrate proximal-to-distal temporal sequencing or intersegmental momentum transfer. Likewise, because kinetic, electromyographic, gaze, and coordination measurements were not obtained, no conclusions can be drawn regarding force or power contribution, trunk stability, visual control, or motor-control quality.

These findings should be read as a descriptive individual biomechanical profile of this athlete's execution under standardised, non-contact laboratory conditions rather than as a normative benchmark or reference model for elite karate performance, and they cannot be generalised across sex, age group, weight category or competitive level. With that caveat, they provide three-dimensional biomechanical evidence that may be of value for comparison in future single- and multi-athlete research on karate punching techniques.

## Data Availability

The raw data supporting the conclusions of this article will be made available by the authors, without undue reservation.
