# Supplementary material for: Angular velocity characteristics and inter-limb asymmetry during jodan-tsuki execution in a world champion female karate athlete: a case study
Source: Front Sports Act Living. 2026 Oct 8;8:1917343. doi: 10.3389/fspor.2026.1917343 (PMC13687389; doi:10.3389/fspor.2026.1917343)
Supplement: Supplementary file 1 [file Datasheet1.pdf]

Supplementary Materials

Table S1. International Competitive Achievements of the World Champion Female Karate Athlete.

Year	Competition	Location	Weight Category	Result
2025	World Karate Championships	Cairo, Egypt	−50 kg	Gold Medal
2025	Asian Karate Championships	Tashkent, Uzbekistan	−50 kg	Gold Medal
2025	CIS Games	Ganja, Azerbaijan	−50 kg	Gold Medal
2025	Islamic Solidarity Games	Riyadh, Saudi Arabia	−50 kg	Silver Medal
2024	Asian Karate Championships	Hangzhou, China	−50 kg	Gold Medal
2024	BRICS Games	Kazan, Russia	−50 kg	Silver Medal

Note. The athlete represented the Uzbekistan National Karate Team and held the title of International-Class Master of Sport of Uzbekistan at the time of testing. The table is provided to document the exceptional competitive status of the participant and support the rationale for the elite-athlete case-study design.

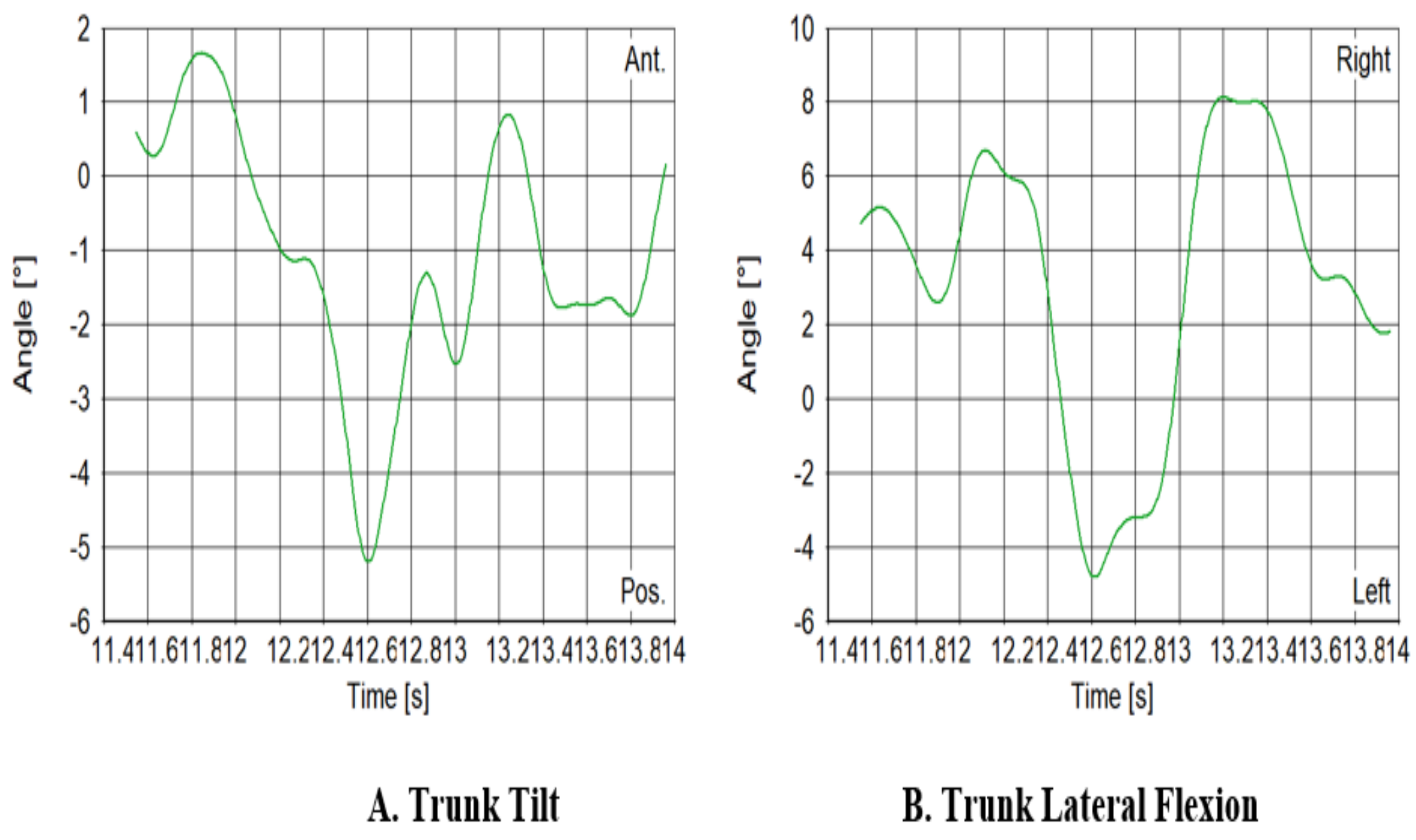

A. Trunk Tilt **B. Trunk Lateral Flexion**

Note for Supplementary Figures S2–S7. The temporal kinematic waveforms presented in Supplementary Figures S2–S7 were obtained from one representative technically successful right-hand Jodan-tsuki trial selected from the 22 trials retained for quantitative analysis. These waveforms are provided for qualitative visualization and do not represent ensemble-averaged profiles across all 22 trials. All quantitative descriptive statistics were calculated using the complete set of 22 technically successful trials.

Figure S2. Temporal profiles of trunk tilt (A) and trunk lateral flexion (B) during right-hand Jodan-tsuki execution in a world champion female karate athlete.

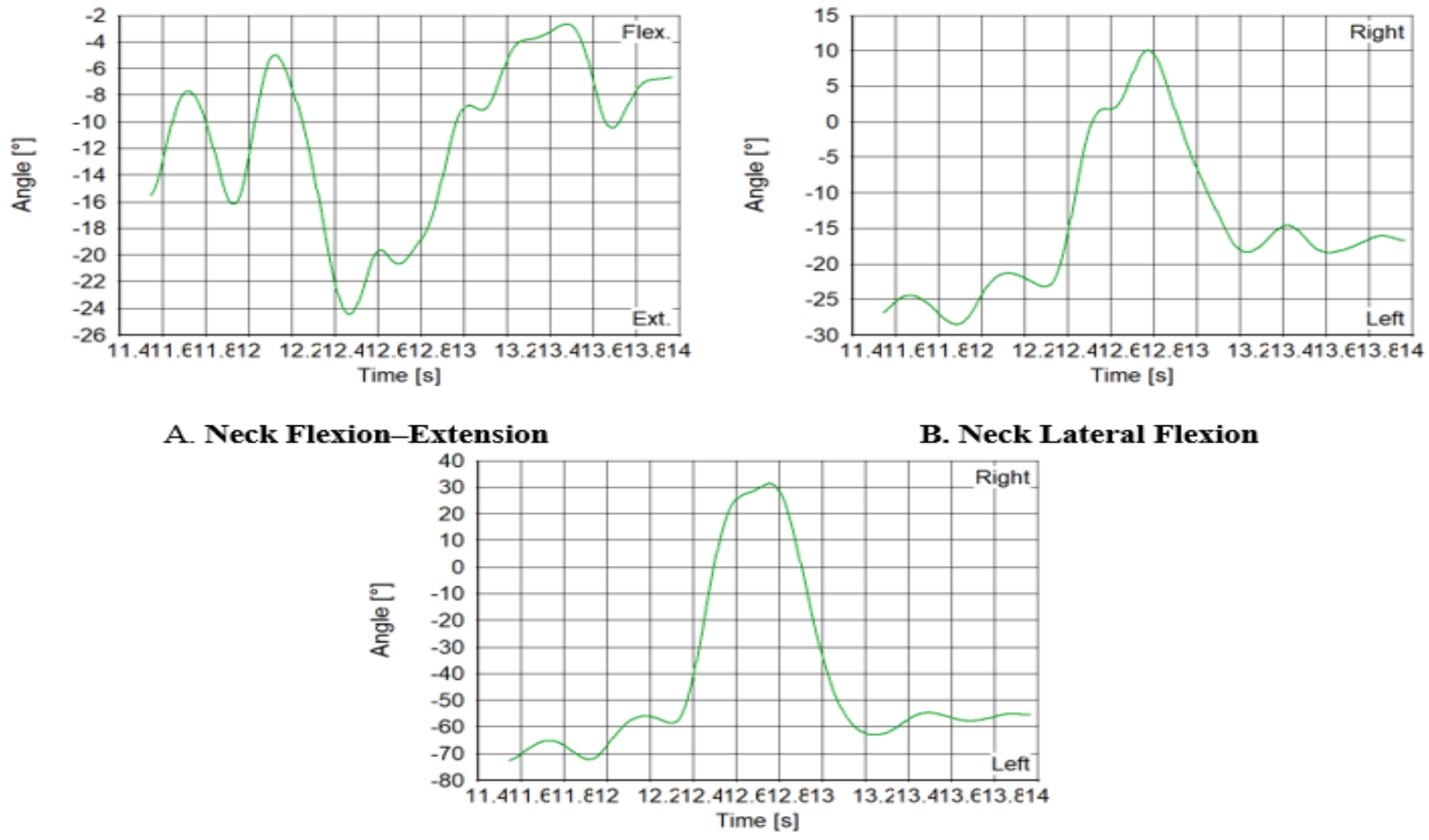

A. Neck Flexion–Extension

B. Neck Lateral Flexion

C. Neck Rotation

Figure S3. Temporal profiles of cervical flexion–extension (A), cervical lateral flexion (B), and cervical rotation (C) during right-hand Jodan-tsuki execution in a world champion female karate athlete.

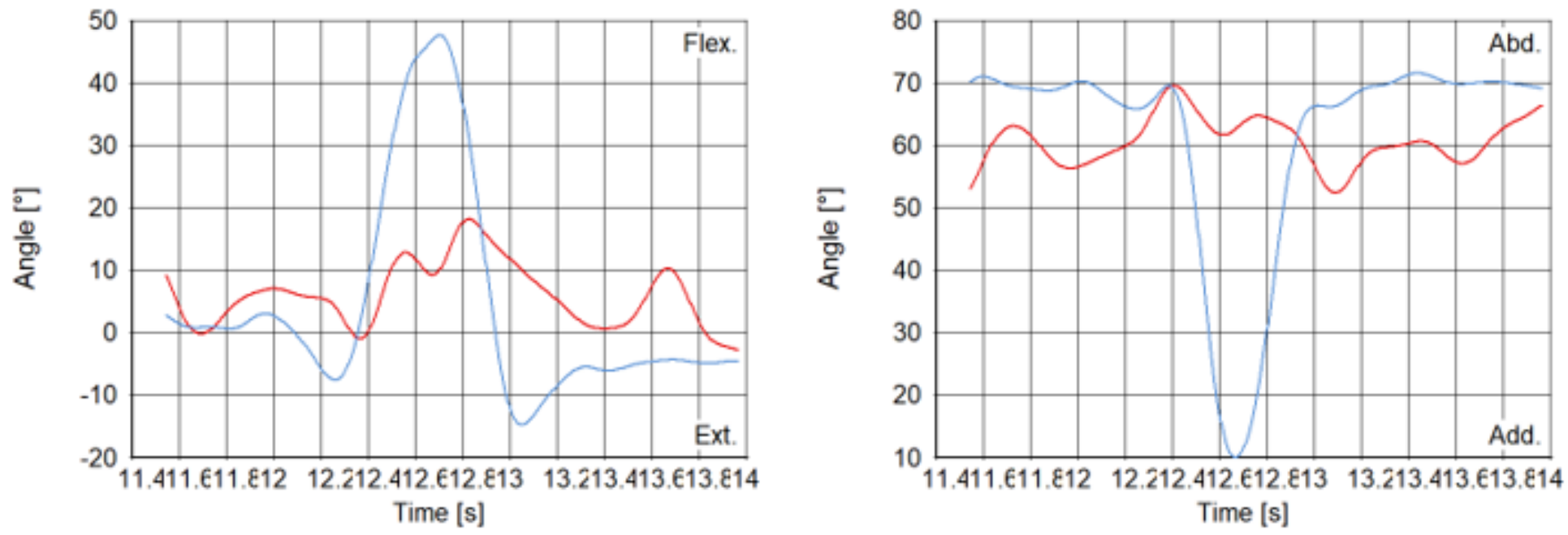

A. Bilateral Shoulder Flexion–Extension

B. Bilateral Shoulder Abduction–Adduction

Figure S4. Bilateral temporal profiles of shoulder flexion–extension (A) and shoulder abduction–adduction (B) during right-hand Jodan-tsuki execution in a world champion female karate athlete. The blue line represents the left shoulder, whereas the red line represents the right shoulder.

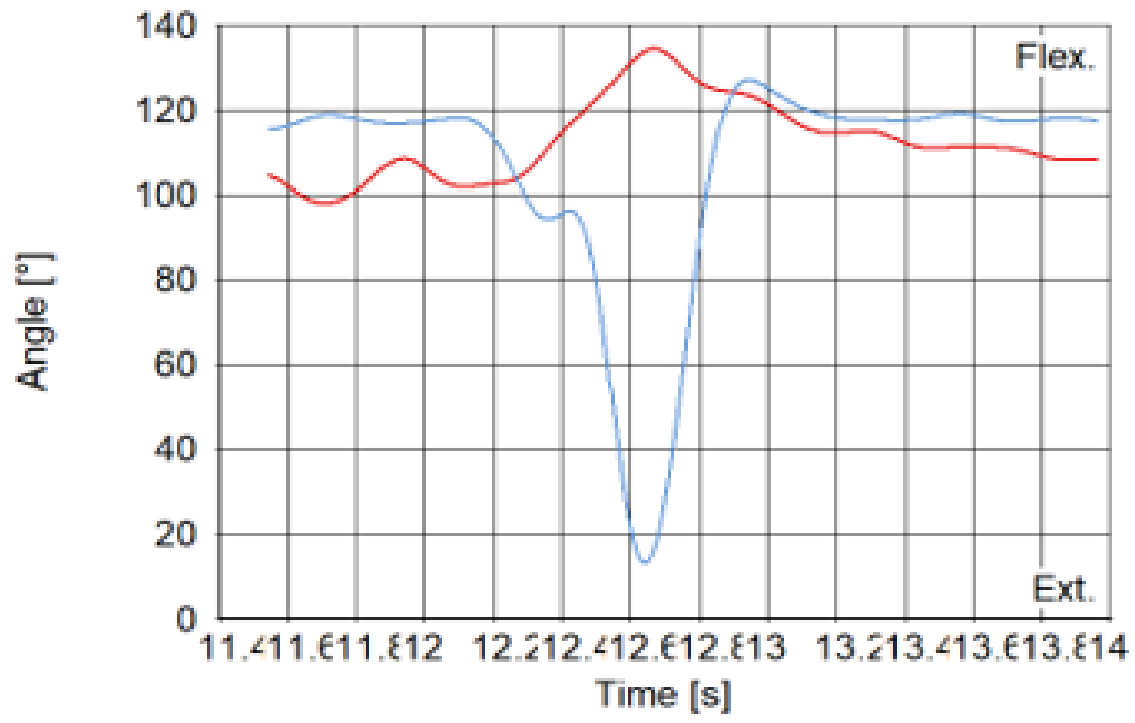

Figure S5. Bilateral temporal kinematic profiles of elbow flexion–extension during right-hand Jodan-tsuki execution in a world champion female karate athlete.

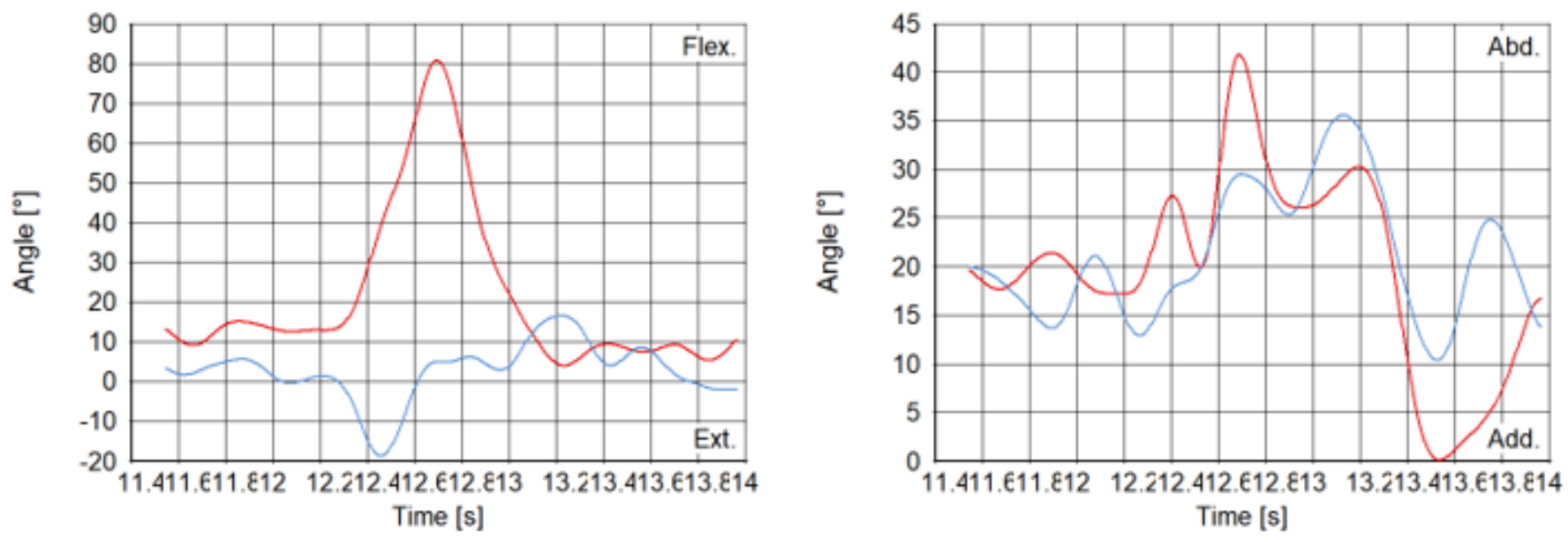

A. Bilateral Hip Flexion–Extension **B. Bilateral Hip Abduction–Adduction**

Figure S6. Bilateral temporal kinematic profiles of hip flexion–extension (A) and hip abduction–adduction (B) during right-hand Jodan-tsuki execution in a world champion female karate athlete.

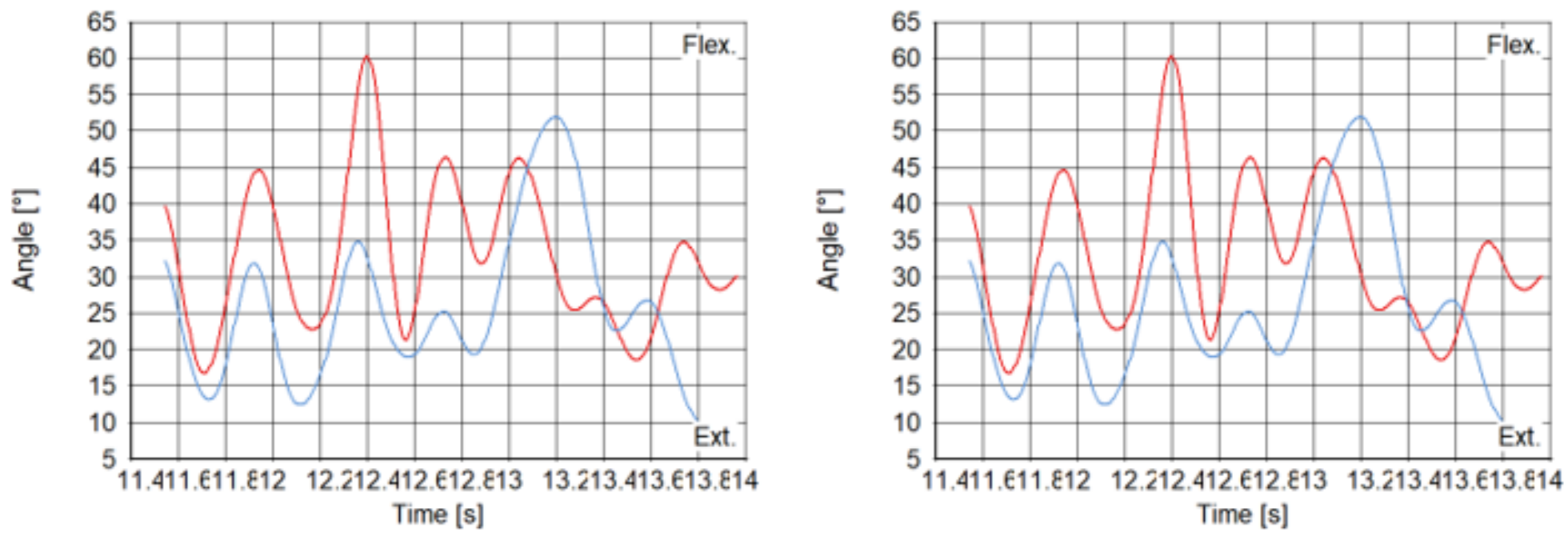

A. Bilateral Knee Flexion–Extension **B. Bilateral Ankle Dorsiflexion–Plantarflexion**

Figure S7. Bilateral temporal kinematic profiles of knee flexion–extension (A) and ankle dorsiflexion–plantarflexion (B) during right-hand Jodan-tsuki execution in a world champion female karate athlete.

Supplementary Table S8. Full Descriptive Statistics for Tables 2–7

*Supplementary Tables S8a–S8f provide the complete descriptive statistics corresponding to Tables 2–7 in the main manuscript, including mean ± standard deviation (SD), 95% confidence intervals (95% CI), coefficients of variation (CV%), and asymmetry indices (AI%) where applicable.*Supplementary Table S8a. Full descriptive statistics (Mean ± SD, 95% CI, CV%) corresponding to main-text Table 2 (Temporal-Spatial Characteristics).

Variable	Mean ± SD	95% CI		CV (%)
		Lower	Upper	
**Cadence (steps·min⁻¹)**	222.22±23.50	211.81	232.63	10.58
**Flight Time (s)**	0.39±0.04	0.37	0.41	10.26
**Support Time (s)**	0.24±0.03	0.23	0.25	12.50
**Step Length (mm)**	171.03±20.10	162.13	179.93	11.75
**Right Flight Time (s)**	0.199±0.020	0.190	0.208	10.05
**Left Flight Time (s)**	0.199±0.021	0.190	0.208	10.55
**COG Vertical Oscillation (mm)**	156.43 ± 15.40	149.61	163.25	9.84
**Right Braking Distance (mm)**	−55.81 ± 6.41	−58.65	−52.97	11.49
**Left Braking Distance (mm)**	−11.70 ± 1.81	−12.50	−10.90	15.47
**Right Propulsion Distance (mm)**	−47.84 ± 5.41	−50.24	−45.44	11.31
**Left Propulsion Distance (mm)**	−9.78 ± 1.40	−10.40	−9.16	14.31
**Right Support Distance (mm)**	609.39 ± 75.40	575.97	642.81	12.37
**Left Support Distance (mm)**	753.08 ± 82.41	716.54	789.62	10.94

Supplementary Table S8b. Full descriptive statistics corresponding to main-text Table 3 (Trunk and Cervical Kinematic Characteristics) — reproduced here unchanged, as Table 3 in the main text retains its full 95% CI columns.

Segment	Variable	Minimum Mean ± SD (°)	95% CI		Maximum Mean ± SD (°)	95% CI	
			Lower	Upper		Lower	Upper
Trunk	Tilt	−6 ± 0.5	−6.22	−5.78	1 ± 0.01	0.996	1.004
	Lateral Flexion	−5 ± 0.4	−5.18	−4.82	8 ± 0.9	7.60	8.40
Neck	Flexion–Extension	−25 ± 2.8	−26.24	−23.76	−3 ± 0.41	−3.18	−2.82
	Lateral Flexion	−29 ± 3.2	−30.42	−27.58	10 ± 1.3	9.42	10.58
	Rotation	1 ± 0.01	0.996	1.004	30 ± 4.2	28.14	31.86

Supplementary Table S8c. Full descriptive statistics (Mean ± SD, 95% CI, AI%) corresponding to main-text Table 4 (Bilateral Upper-Limb ROM Characteristics).

Variable	Left Mean ± SD (°)	95% CI		Right Mean ± SD (°)	95% CI		AI (%)
		Lower	Upper		Lower	Upper	
Shoulder Flexion ROM	19 ± 2.3	17.98	20.02	62 ± 6.7	59.03	64.97	106.2
Shoulder Abduction ROM	12 ± 1.4	11.38	12.62	11 ± 1.4	10.38	11.62	8.7
Elbow Flexion ROM	36 ± 4.2	34.14	37.86	14 ± 1.6	13.29	14.71	88.0

Supplementary Table S8d. Full descriptive statistics corresponding to main-text Table 5 (Bilateral Lower-Limb ROM Characteristics).

Variable	Left Mean ± SD (°)	95% CI		Right Mean ± SD (°)	95% CI		AI (%)
		Lower	Upper		Lower	Upper	
Hip Flexion ROM	80 ± 8.4	76.28	83.72	35 ± 4.2	33.14	36.86	78.3
Hip Abduction ROM	41 ± 5.2	38.70	43.30	25 ± 3.2	23.58	26.42	48.5
Knee Flexion ROM	43 ± 4.8	40.87	45.13	39 ± 4.2	37.14	40.86	9.8
Ankle Dorsiflexion–Plantarflexion ROM	52 ± 5.9	49.39	54.61	47 ± 5.4	44.61	49.39	10.1

Supplementary Table S8e. Full descriptive statistics corresponding to main-text Table 6 (Bilateral Asymmetry Index).

Variable	Left Mean ± SD (°)	95% CI		Right Mean ± SD (°)	95% CI		AI (%)
		Lower	Upper		Lower	Upper	
Shoulder Flexion	19 ± 1.8	18.20	19.80	62 ± 6.7	59.03	64.97	106.2
Shoulder Abduction	12 ± 1.3	11.42	12.58	11 ± 1.3	10.42	11.58	8.7
Elbow Flexion	36 ± 5.2	33.70	38.30	14 ± 1.5	13.34	14.66	88.0
Hip Flexion	80 ± 8.2	76.37	83.63	35 ± 4.1	33.18	36.82	78.3
Hip Abduction	41 ± 4.5	39.01	42.99	25 ± 3.2	23.58	26.42	48.5
Knee Flexion	43 ± 5.2	40.70	45.30	39 ± 4.2	37.14	40.86	9.8
Ankle Flexion	52 ± 5.6	49.52	54.48	47 ± 5.3	44.65	49.35	10.1

Supplementary Table S8f. Full descriptive statistics (Mean ± SD, 95% CI, CV%) corresponding to main-text Table 7 (Peak Segmental Angular Velocity Characteristics).

Segment	Variable	Mean ± SD (°·s⁻¹)	95% CI		CV (%)
			Lower	Upper	
Pelvis	Rotation	58.77 ± 6.20	56.02	61.52	10.55
Hip	Right flexion–extension	33.36 ± 4.10	31.54	35.18	12.29
	Left flexion–extension	51.67 ± 6.50	48.79	54.55	12.58
	Right abduction–adduction	73.78 ± 8.40	70.06	77.50	11.39
	Left abduction–adduction	42.32 ± 5.30	39.97	44.67	12.52
Knee	Right flexion–extension	153.97 ± 16.24	146.77	161.17	10.55
	Left flexion–extension	174.39 ± 18.20	166.32	182.46	10.44
Ankle	Right flexion–extension	237.53 ± 24.02	226.88	248.18	10.11
	Left flexion–extension	193.97 ± 21.10	184.62	203.32	10.88
Shoulder	Right flexion–extension	141.40 ± 15.02	134.74	148.06	10.62
	Left flexion–extension	94.48 ± 9.82	90.13	98.83	10.39
	Right flexion–extension (vertical reference)	137.03 ± 15.20	130.29	143.77	11.09
	Left flexion–extension (vertical reference)	53.18 ± 6.21	50.43	55.93	11.68
	Right abduction–adduction	168.45 ± 18.20	160.38	176.52	10.80
	Left abduction–adduction	73.45 ± 8.20	69.81	77.09	11.16
Elbow	Right flexion–extension	351.42 ± 38.12	334.53	368.31	10.85
	Left flexion–extension	67.48 ± 7.80	64.02	70.94	11.56
